# Global epidemiology of drug resistance after failure of WHO recommended first-line regimens for adult HIV-1 infection: a multicentre retrospective cohort study

**DOI:** 10.1016/S1473-3099(15)00536-8

**Published:** 2016-05

**Authors:** The TenoRes Study Group

## Abstract

**Background:**

Antiretroviral therapy (ART) is crucial for controlling HIV-1 infection through wide-scale treatment as prevention and pre-exposure prophylaxis (PrEP). Potent tenofovir disoproxil fumarate-containing regimens are increasingly used to treat and prevent HIV, although few data exist for frequency and risk factors of acquired drug resistance in regions hardest hit by the HIV pandemic. We aimed to do a global assessment of drug resistance after virological failure with first-line tenofovir-containing ART.

**Methods:**

The TenoRes collaboration comprises adult HIV treatment cohorts and clinical trials of HIV drug resistance testing in Europe, Latin and North America, sub-Saharan Africa, and Asia. We extracted and harmonised data for patients undergoing genotypic resistance testing after virological failure with a first-line regimen containing tenofovir plus a cytosine analogue (lamivudine or emtricitabine) plus a non-nucleotide reverse-transcriptase inhibitor (NNRTI; efavirenz or nevirapine). We used an individual participant-level meta-analysis and multiple logistic regression to identify covariates associated with drug resistance. Our primary outcome was tenofovir resistance, defined as presence of K65R/N or K70E/G/Q mutations in the reverse transcriptase (*RT*) gene.

**Findings:**

We included 1926 patients from 36 countries with treatment failure between 1998 and 2015. Prevalence of tenofovir resistance was highest in sub-Saharan Africa (370/654 [57%]). Pre-ART CD4 cell count was the covariate most strongly associated with the development of tenofovir resistance (odds ratio [OR] 1·50, 95% CI 1·27–1·77 for CD4 cell count <100 cells per μL). Use of lamivudine versus emtricitabine increased the risk of tenofovir resistance across regions (OR 1·48, 95% CI 1·20–1·82). Of 700 individuals with tenofovir resistance, 578 (83%) had cytosine analogue resistance (M184V/I mutation), 543 (78%) had major NNRTI resistance, and 457 (65%) had both. The mean plasma viral load at virological failure was similar in individuals with and without tenofovir resistance (145 700 copies per mL [SE 12 480] versus 133 900 copies per mL [SE 16 650; p=0·626]).

**Interpretation:**

We recorded drug resistance in a high proportion of patients after virological failure on a tenofovir-containing first-line regimen across low-income and middle-income regions. Effective surveillance for transmission of drug resistance is crucial.

**Funding:**

The Wellcome Trust.

## Introduction

More than 35 million people worldwide are living with HIV-1.^1^ There is no effective vaccine and therefore control of the HIV pandemic relies heavily on combination antiretroviral therapy (cART). WHO treatment guidelines for adult HIV-1 infection recommend the nucleotide reverse-transcriptase inhibitor (NRTI) tenofovir for first-line ART, in combination with lamivudine or emtricitabine and the non-nucleoside reverse-transcriptase inhibitor (NNRTI) efavirenz.^2^ Older NRTIs such as the thymidine analogue drugs are being replaced by tenofovir and the NNRTI nevirapine, although mentioned in WHO guidelines, is being phased out from first-line regimens.^2^

The global scale-up of cART has now reached 15 million treated individuals.^1^ The administration of cART at the time individuals with HIV-1 are initially diagnosed prevents immunological deterioration as early as possible and interrupts the spread of HIV-1 from newly diagnosed individuals.^3^ This strategy, referred to as treatment as prevention, is being studied especially in high-incidence regions and nearly always includes the use of first-line tenofovir-containing ART regimens. Likewise, the strategy of pre-exposure prophylaxis (PrEP) depends entirely on the administration of tenofovir or tenofovir and emtricitabine to uninfected individuals at high risk of HIV-1 infection.^4^

In individuals receiving tenofovir, HIV-1 develops phenotypically and clinically significant resistance usually as a result of one mutation at position 65 (lysine to arginine; K65R) in the reverse transcriptase (*RT*) gene.^5^ Data from clinical trials and cohorts in high-income settings using tenofovir combined with NNRTI have reported low prevalence of tenofovir resistance at viral failure,^6^, ^7^, ^8^ in stark contrast with reports from low-income and middle-income countries where prevalence seems to be much higher.^9^, ^10^ Similarly, high-level resistance to NNRTI and the cytosine analogue component (emtricitabine and lamivudine) arise through changes to one aminoacid, which suggests a low genetic barrier to resistance for these drugs as well. In view of the pivotal role of tenofovir-containing ART as both treatment and prophylaxis, and the striking potential for drug resistance, we did a global assessment of drug resistance after virological failure with first-line tenofovir-containing ART.

**Research in context**

**Evidence before this study**

We searched PubMed for studies of the prevalence of tenofovir resistance after failure of first-line antiretroviral therapy with efavirenz or nevirapine (non-nucleoside reverse-transcriptase inhibitors [NNRTIs]) in patients with HIV-1, published between January, 1999, and June, 2015, using the search terms “HIV” AND “tenofovir” AND “resistance”. We identified studies done in untreated adults (age >15 years) in which either efavirenz or nevirapine was combined with tenofovir and either emtricitabine or lamivudine as first line antiretroviral therapy. Several studies reported resistance data for tenofovir when the drug was started after initial use of stavudine or zidovudine; these studies were not reviewed further. We also excluded studies that reported tenofovir use without NNRTI because standard first-line antiretroviral therapy under a public health approach is based on NNRTI in adults.

We identified randomised controlled trials and a meta-analysis comparing NNRTI with protease inhibitors, in combination with tenofovir, which reported resistance data. Patients in high-income settings reported tenofovir resistance in 0–25% of virological failures and those in sub-Saharan Africa in 28–50%. The only other prospective study in sub-Saharan Africa was PASER-M, and was limited by few resistance data for patients given tenofovir plus NNRTI-based combination antiretroviral therapy (cART). The remaining studies were largely from South Africa and reported a wide range of prevalence (between 23% and 70%) of tenofovir resistance after virological failure. In west Africa, one study reported that 57% of virological failures were tenofovir resistant in a very small sample of 23 patients. Although aforementioned studies also reported NNRTI and cytosine analogue resistance, they were unable to quantify to what extent tenofovir resistance was a marker for high-level compromise of the regimen. We found no studies that specifically reported resistance data for patients given first-line tenofovir in east Africa. No study reported resistance data from more than one continent, and none seemed adequately powered to establish the effect of co-administered reverse-transcriptase inhibitors on the emergence of tenofovir resistance.

**Added value of this study**

This study reports the most comprehensive assessment of HIV-1 drug resistance after scale-up of first-line WHO recommended tenofovir-based antiretroviral regimens, showing that tenofovir resistance is surprisingly common in patients with treatment failure across many studies in all low-income regions. Importantly, these individuals also have notable resistance to other drugs in their regimen, leading to almost complete compromise of combination treatment. Challenging current perceptions in the specialty, our findings show that tenofovir resistant viruses have substantial transmission potential. Furthermore, our results show that viral strain affects tenofovir resistance in Europe but is not the main driver for resistance in viruses circulating in sub-Saharan Africa. Newly identified risk factors for resistance to tenofovir and NNRTI drugs include pre-treatment CD4 cell count (but not viral load) and co-administered antiretrovirals.

**Implications of all the available evidence**

Improvements in the quality of HIV care and viral load monitoring could mitigate the emergence and spread of tenofovir resistance, thereby prolonging the lifetime of tenofovir-containing regimens for both treatment and prophylaxis. Surveillance of tenofovir and NNRTI resistance should be a priority both in untreated and treated populations.

## Methods

### Study population and design

The TenoRes collaboration comprises adult HIV treatment cohorts and clinical trials from Europe, Latin and North America, sub-Saharan Africa, and Asia. Cohorts and trials were identified by RWS and RKG as those known to do genotypic resistance testing through previous collaborations, the WHO HIV Drug Resistance Network, and through the International HIV Drug Resistance Workshop. Moreover, we did a systematic review using the keywords “HIV”, AND “tenofovir” AND “resistance” in PubMed for articles published between January, 1999, and June, 2015. We identified 44 studies suitable for the reported analysis after applying the following inclusion criteria: documented virological failure after first-line ART comprising tenofovir plus either lamivudine or emtricitabine plus either efavirenz or nevirapine (virological failure was defined by local viral load thresholds or surveillance protocols); a successful resistance test result associated with virological failure of cART; tenofovir-based ART for at least 4 months before virological failure; and absence of thymidine analogue mutations at resistance testing (appendix). Exclusion criteria were: studies reporting resistance data after tenofovir that was started after initial use of stavudine or zidovudine; and studies reporting tenofovir use without NNRTI. Data were extracted and harmonised by RWS, RKG, MT, and JG and stored in a central database.

We collected individual-level data for a predefined set of covariates: age at first-line ART initiation, sex, frequency of viral load monitoring (number of tests per year), urban versus rural setting for HIV clinics, viral load threshold for virological failure and genotyping, co-administered antiretrovirals, duration of treatment, viral load and CD4 cell count before the start of first-line ART (baseline) and at time of viral failure, and resistance mutations based on the Stanford HIV Drug Resistance Database.

### Statistical analysis

Our primary outcome was tenofovir resistance, defined as presence of K65R/N or K70E/G/Q mutations in the RT gene. Our secondary outcomes were resistance to first generation NNRTI (efavirenz and nevirapine), defined as specific mutations at aminoacid positions 100, 103, 106, 108, 181, 188, 190, and 225,^11^ and cytosine analogue resistance, defined as presence of M184V/I. Our main exposures of interest were baseline CD4 cell count (<100 *vs* ≥100 cells per μL), baseline viral load (<100 000 *vs* ≥100 000 copies HIV-1 RNA per mL; this cutoff was chosen because of findings from previous studies^12^), nevirapine versus efavirenz, and lamivudine versus emtricitabine. For our primary analysis, we estimated the odds ratios (ORs) for tenofovir resistance within each study before pooling estimates across studies using a random-effects meta-analysis with DerSimonian-Laird weighting and estimates of heterogeneity taken from the Mantel-Haenszel model. We chose this method to ensure that we only compared patients in the same study and country, thereby minimising confounding by differences in care at the study or country level. Findings were not sensitive to the choice of method used for the meta-analysis (ie, fixed or random effects). We also used a continuity correction of 0·5 for counts of 0, although findings were not sensitive to this choice.

We did sensitivity analyses to investigate whether associations changed when adjusted for possible confounders. Because of the sparseness of data in many studies, we were unable to adjust within-study associations for potential confounders. Instead, we did additional analyses using logistic regression models with a random effect at study level to estimate associations before and after adjustment for possible confounders in a common subset of participants. To build the adjusted model, we included each of our main exposures and HIV subtype. We also considered for inclusion individual-level information about age, sex, year of treatment initiation, and length of time on tenofovir, but rejected these covariates because of a lack of any univariate association with tenofovir resistance. We chose to only use these models for working out the likely extent of confounding, because estimated associations from these models are partly derived from between-study comparisons.

To clarify whether the association between baseline CD4 or baseline viral load and tenofovir resistance was linear (ie, followed a dose-response pattern), we categorised participants into four categories based on baseline CD4 cell count (<100, 100–200, 201–300, >300 cells per μL reference category) or baseline viral load (<25 000 [reference]; 25 001–100 000; 100 001–300 000; >300 000 copies HIV-1 RNA per mL). We assessed associations by plotting the estimated OR against the mean level of baseline CD4 (or baseline viral load), in a random-effects logistic regression model adjusted for region, co-administered drugs, and baseline viral load (or baseline CD4).

To assess the potential transmissibility of mutant viruses, we graphically compared the distribution of plasma HIV-1 RNA concentrations of patients from the same study with and without tenofovir resistance.

We did not use multiple imputation to adjust for missing data because most missing data were the result of a lack of availability at the study level. Instead, we restricted analyses to the subset of participants with information available about all relevant covariates for each specific analysis. The appendix presents the amount of missing data and which studies contributed towards specific analyses. We used Stata (version 11.2) for all analyses.

### Role of the funding source

The funders of the study had no role in study design, data collection, data analysis, data interpretation, or writing of the report. RKG and JG had full access to all the data in the study and had final responsibility for the decision to submit for publication.

## Results

**Figure 1:**
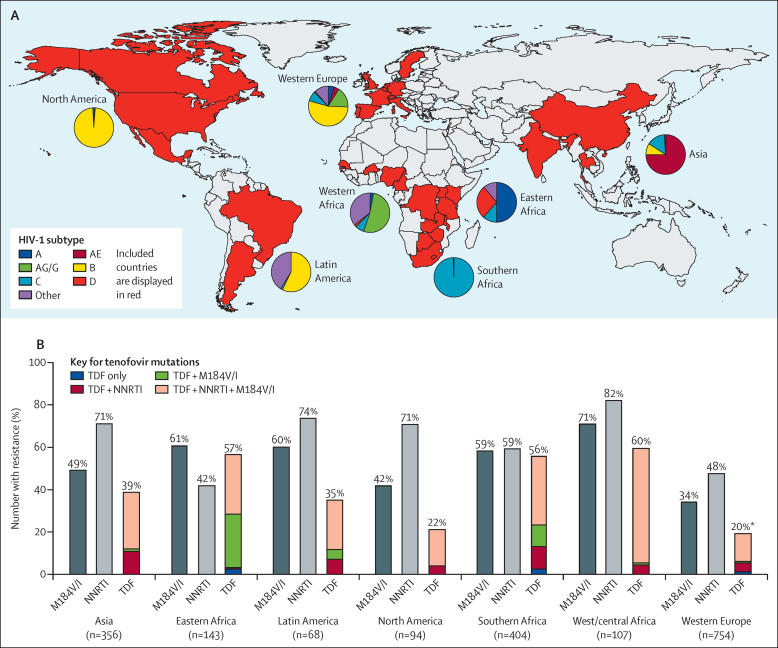
(A) Countries contributing data to resistance analysis and HIV-1 subtype distribution, (B) prevalence of drug resistance by mutation and by region NNRTI=non-nucleotide reverse-transcriptase inhibitor. TDF=tenofovir disoproxil fumarate. *24% (n=462) of participants had tenofovir resistance when genotypes from viral load >1000 copies HIV-1 RNA per mL were considered.

The TenoRes collaboration included 1926 individuals from 36 countries (figure 1 and appendix). Table 1 summarises the median size and year of ART initiation for the cohorts comprising the collaboration. Viral load monitoring was done in about 50% of the cohorts including nearly all of cohorts from upper-income regions and from a small proportion of the cohorts in low-income and middle-income countries (appendix shows income status for each cohort; table 1).

The region-level pre-ART median CD4 cell count ranged from 44 to 104  cells per μL in sub-Saharan Africa, Asia, and Latin America (table 2). As expected, in north America pre-ART median CD4 cell count was 144 cells per μL and 190  cells per μL in Europe. The proportion of individuals using emtricitabine (*vs* lamivudine) and efavirenz (*vs* nevirapine) varied significantly by region. Emtricitabine was used significantly more than lamivudine in Europe, North America, and west and central Africa, and efavirenz was used significantly more than nevirapine in all regions apart from east and west and central Africa. The median duration of ART ranged from 11 to 26 months. Pre-treatment viral load ranged between 4·80 and 5·58 log copies per mL and was significantly higher in eastern and western and central Africa and Latin America than the other regions (table 2).

Crude prevalence of tenofovir resistance in patients with treatment failure was highest in low-income and middle-income regions (figure 1). Prevalence of cytosine analogue resistance (M184V/I) was highest in sub-Saharan Africa and Latin America and lowest in western Europe. By contrast, resistance to NNRTI did not show this pattern (figure 1). Furthermore, the M184V/I mutation was less common than NNRTI resistance across all regions except in eastern Africa. Of the 700 patients with tenofovir resistance in the dataset, 457 (65%) had resistance to both remaining drugs. Participants with tenofovir resistant viruses were likely to be resistant to one or both accompanying drugs and therefore have profound compromise of their regimen, as compared with those without tenofovir resistance (figure 1).

**Figure 2:**
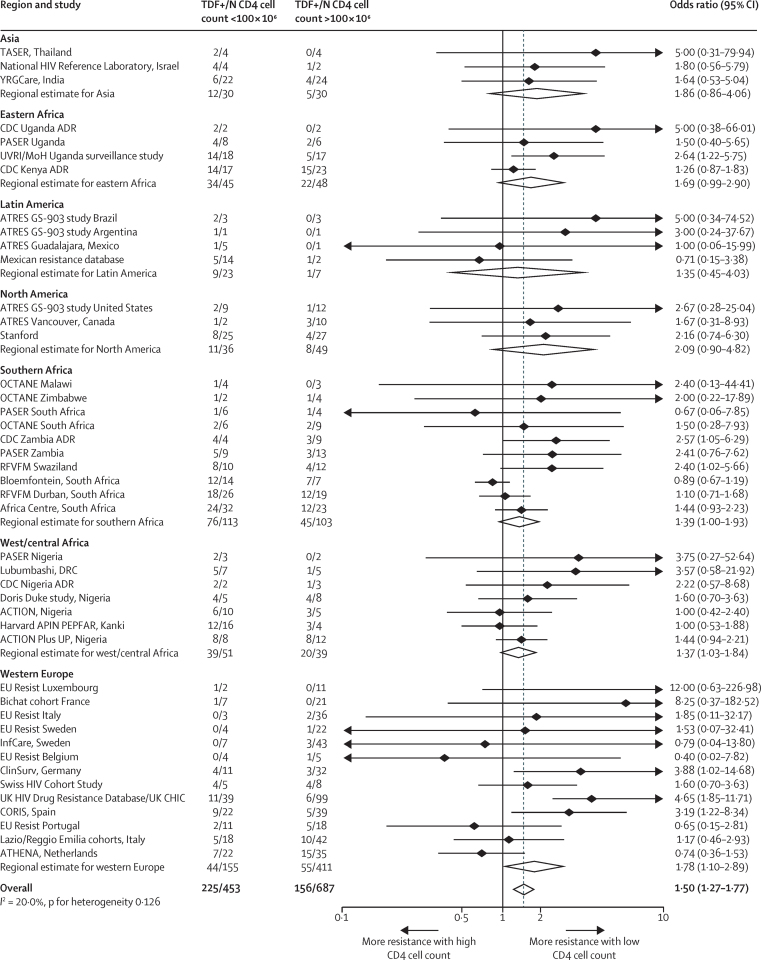
Pooled odds ratios for tenofovir resistance after viral failure for baseline CD4 cell count <100 *vs* ≥100 × 10^6^ cells per μL TDF+ denotes presence of tenofovir resistance. TDF=tenofovir disoproxil fumarate.

Low baseline CD4 cell count was consistently associated with a higher prevalence of tenofovir resistance across regions. The pooled OR for tenofovir in individuals with a CD4 cell count of less than 100 cells per μL versus 100  cells per μL was 1·50 (95% CI 1·27–1·77; figure 2). By contrast, a high baseline viral load was only associated with a small, not significant increase in tenofovir resistance (OR for viral load ≥100 000 copies per mL *vs* <100 000 copies per mL was 1·17, 95% CI 0·94–1·44; appendix). We compared tenofovir resistance by use of co-administered antiretrovirals with tenofovir as first-line therapy. Use of lamivudine rather than emtricitabine (NRTIs) was associated with a higher prevalence of tenofovir resistance (OR 1·48, 95% CI 1·20–1·82), as was use of the NNRTI nevirapine rather than efavirenz (OR 1·46, 1·28–1·67; appendix). Subgroup analysis showed that as well as associations being consistent across regions, they were also generally similar across a range of study settings (appendix), although there was some evidence of a greater effect size of baseline CD4 when efavirenz was co-administered with tenofovir, as compared with nevirapine.

**Figure 3:**
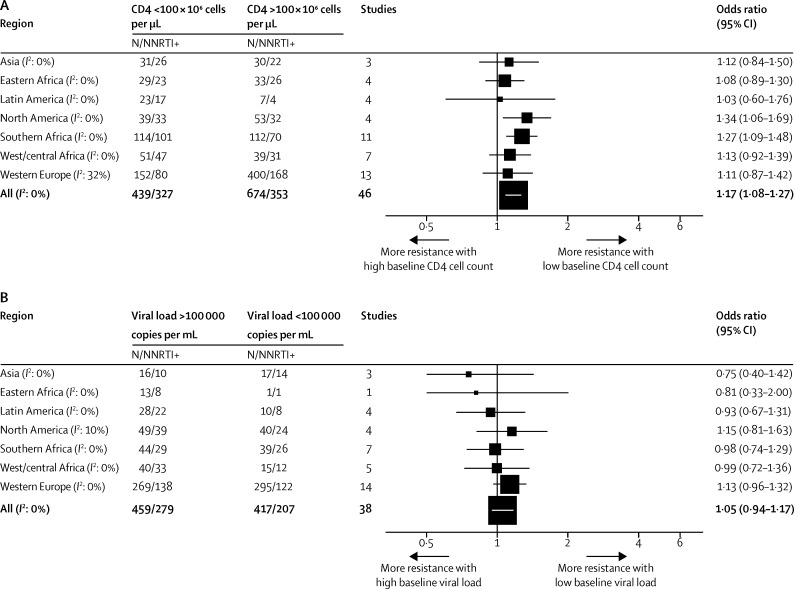
Odds ratios for NNRTI resistance for (A) baseline CD4 cell count <100 *vs* ≥100 cells per μL, (B) viral load ≥100 000 *vs* <100 000 copies HIV-1 RNA per mL NNRTI=non-nucleotide reverse-transcriptase inhibitor.

When considering the effect of baseline CD4, baseline viral load (figure 3), and co-administered antiretrovirals (appendix) on cytosine analogue and NNRTI resistance, we noted that the magnitude of associations were smaller than those recorded for tenofovir resistance.

We also assessed the relation between viral subtype C on acquisition of tenofovir resistance. We restricted this analysis to western European studies in view of the consistent standard of care available in this region and relatively lower level of subtype diversity in other regions (figure 1A). We also limited the comparison to subtypes found in immigrant populations to minimise bias due to socioeconomic factors (thereby excluding subtype B infections mainly recorded in participants born in western Europe). Tenofovir resistance was higher in subtype C compared with non-C, non-B infections with a pooled OR of 2·44 (1·66–3·59).

As a sensitivity analysis we studied risk factors for tenofovir resistance using univariate (adjusted only for region) and multivariate logistic regression analyses (appendix). We noted a dose-response relationship for baseline CD4, which was not markedly altered by adjustment for baseline viral load, viral subtype, or type of co-administered drug used (appendix). Baseline viral load of 100 000 or more copies of HIV-1 RNA per mL was not significantly associated with tenofovir resistance (OR 1·31, 95% CI 0·91–1·91) and we noted no clear trend across increasing viral loads (appendix). Adjustment for several risk factors also had little effect on associations with tenofovir resistance of emtricitabine versus lamivudine and nevirapine versus efavirenz.

**Figure 4:**
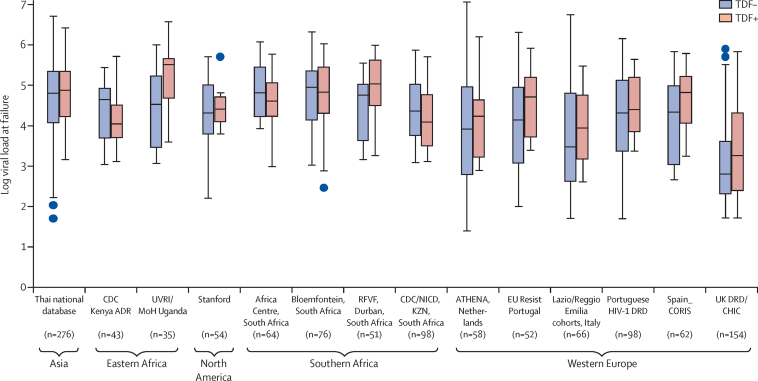
Boxplot of log viral load by presence (TDF-positive) or absence (TDF-negative) of tenofovir resistance at viral failure in studies with at least ten patients with TDF resistance and a viral load measurement at treatment failure We restricted to studies with at least ten TDF-resistant mutations to help with graphical clarity, although the pattern of similar distributions of failure viral load in the presence or absence of TDF resistance was true for all studies. TDF=tenofovir disoproxil fumarate. Blue dots represent outliers.

Finally, we compared the viral load at treatment failure in the presence and absence of tenofovir-associated mutations. The mean plasma viral load at treatment failure was not different in the presence or absence of tenofovir associated mutations (145 700 copies HIV RNA per mL [SE 12 480] *vs* 133 900 copies [SE 16 650]; p=0·626; figure 4 shows the within-study viral load by region). These results did not change when analysis was restricted to individuals who had evidence of the K65R mutation, either with or without M184V/I (appendix). Mutations at aminoacids K65 and M184 in the RT gene have been associated with suboptimum replication.^13^

## Discussion

Our study has three main findings relating to the prevalence, risk factors for, and transmissibility of tenofovir resistance. First, we noted that levels of tenofovir resistance in individuals with viral failure ranged from 20% in Europe to more than 50% in sub-Saharan Africa. Second, a CD4 cell count of less than 100 cells per μL, treatment with nevirapine rather than efavirenz, and treatment with lamivudine rather than emtricitabine, were consistently associated with a 50% higher odds of tenofovir resistance in those with viral failure. Third, we noted that in patients with viral failure, viral loads were similar in the presence or absence of tenofovir resistance.

Our findings are important in view of the fact that following WHO recommendations,^2^ tenofovir is replacing thymidine analogues (zidovudine and stavudine) as part of the NRTI backbone in first-line regimens in resource-limited settings. Every drug in these regimens can be compromised by one aminoacid mutation, and the combination therapy is therefore potentially fragile. In view of the crucial role of tenofovir-containing ART in both treatment and prevention of new infections, restriction of drug resistance in high-burden settings is of paramount importance. Understanding how common tenofovir resistance is, and how and why it varies, is key to its prevention. Although our risk factors are only associated with a modest 50% increase in odds, this translates to a roughly 10% increase in resistance in those who fail when the overall tenofovir resistance prevalence is about 50% (as recorded in sub-Saharan Africa).

We hypothesise that the regional differences in tenofovir resistance are due to the frequency of viral load monitoring with close patient follow-up and feedback of results. For example, although viral load monitoring is not routinely done in most low-income and middle-income countries, in high-income countries viral load is tested three to four times per year with close patient follow-up and adherence support. Such an approach is likely to lead to earlier detection of viral failure, before selection of drug resistance mutations against tenofovir has occurred.^14^ This view is supported by the uncommon detection of drug resistance mutations in specimens with low viral load (400–1000 copies per mL) from patients given tenofovir in both high-income settings (figure 1; see higher prevalence of tenofovir resistance where viral load >1000 copies per mL is used as threshold in western Europe)^15^ and sub-Saharan Africa (Chunfu Yang, Centres for Disease Control, Atlanta, GA, USA, personal communication). Tenofovir resistance could be limited by viral load monitoring,^16^ with rapid feedback to clinicians followed by adherence counselling to preserve first line, or switch to second line when this approach fails. Furthermore, pre-ART (baseline) resistance testing for key NNRTI mutations could potentially protect against tenofovir resistance by avoiding use of partly active treatment regimens. In our report, transmitted NNRTI resistance was low in the regions studied (<10%),^17^ and therefore not likely to be a major driver of wide variation in drug resistance across income settings.

Other factors that vary geographically could also affect success of ART and should be noted. Treatment failure is associated not only with drug resistance, but also side-effects. Efavirenz is associated with CNS side-effects such as sleep disturbance and is associated with treatment discontinuation.^18^ Furthermore, drug stock-outs and other indicators of quality of HIV services that have shown geographic variation would also predispose to treatment failure.^19^ The issue of regional variation in adherence levels has received considerable attention, with data from several studies suggesting that adherence is not worse in sub-Saharan Africa compared with North America.^20^, ^21^

With regards to increased tenofovir resistance in individuals with low baseline CD4 counts, this finding is consistent with results from the ACTG 5202 trial^22^ suggesting higher frequency of RT mutations in patients given ART with low CD4 cell counts, and offer a benefit of CD4 cell count measurement after diagnosis of HIV infection beyond establishing prophylaxis against opportunistic infections.^23^ Lamivudine warrants further study in first-line regimens in view of data presented in our study and the conflicting reports regarding virological efficacy of lamivudine versus emtricitabine.^24^, ^25^, ^26^ Of note, the differences between lamivudine and emtricitabine might become less important in high-income regions where implementation of the second generation integrase inhibitor dolutegravir occurs, in view of the fact that this agent has not been associated with any cytosine analogue resistance at virological failure.^27^

Viral load has been associated with transmission risk.^28^ Despite evidence for diminished replication of tenofovir resistant viruses (containing the K65R mutation in the RT gene) in vitro, we noted similar viral loads in participants with and without tenofovir resistance. Therefore, there might be substantial potential for onward transmission to uninfected individuals,^29^ despite little evidence of K65R transmission up to now.^30^ This finding reinforces the need for drug resistance surveillance activities in both untreated and treated HIV-positive individuals.

There are several important limitations of our study. First, because we only included patients with virological failure related to existing study cohorts,^1^ our estimates of the prevalence of tenofovir resistance might not be representative in certain high-burden regions. Although this situation might have biased our findings on absolute prevalences of tenofovir resistance, it is unlikely to have affected associations with baseline CD4 or co-administered drugs. Second, we only included patients at failure so were unable to assess overall rates of tenofovir resistance in all patients starting first-line treatment. We used this method because many of the contributing studies had no clear denominator, especially those done in resource-limited settings. However, extensive WHO-led analysis reported that 15–35% (on treatment *vs* intention to treat) of patients in sub-Saharan Africa have virological failure by 12 months.^31^ Therefore, using a conservative 50% prevalence of tenofovir resistance at failure from our analysis, we suggest that it is likely that 7·5–17·5% of individuals given tenofovir plus cytosine analogue plus efavirenz will develop tenofovir resistance within 1 year of treatment initiation under present practices in sub-Saharan Africa.

Third, our findings on risk factors for tenofovir resistance were derived from an unadjusted meta-analysis involving very different study populations. Although this enhances the generalisability of results, it has the potential to lead to biased comparisons. However, we took measures to minimise biases. We exclusively used within-study and within-country comparisons for our primary analyses, thereby ensuring that comparisons were for participants undergoing similar treatment monitoring practices. We tested associations between risk factors and found that they were generally weak. For example, baseline CD4 cell count and viral load were only weakly associated with one another and neither was strongly associated with type of co-administered drug. Additionally, we undertook sensitivity analyses, which suggested that adjustment for other covariates had minimum effect on estimated associations. Lastly, our data tended to be consistent with previous studies—eg, our findings of higher resistance in subtype C patients are consistent with in-vitro data suggesting subtype C viruses are more susceptible to developing the K65R mutation.^32^

Fourth, despite our analysis being the largest drug resistance study ever undertaken after failure of first-line tenofovir-containing cART, patient numbers were somewhat limited by the slow uptake of tenofovir-based regimens in west and central Africa, eastern Europe, and Asia (in particular China and Russia), and information about baseline viral load in these settings was uncommon. As a result, European countries, Thailand, and South Africa contributed substantially to the analysis.

In summary, extensive drug resistance emerges in a high proportion of patients after virological failure on a tenofovir-containing first-line regimen across low-income and middle-income regions. Optimisation of treatment programmes and effective surveillance for transmission of drug resistance is therefore crucial.

Correspondence to: Dr Ravindra K Gupta, UCL, Department of Infection, London WC1E 6BT, UK **ravindra.gupta@ucl.ac.uk**

or

Prof Robert W Shafer, Department of Medicine, Stanford University, Stanford, CA 94305, USA **rshafer@stanford.edur**

## Figures and Tables

**Table 1:** Characteristics of resistance studies included in analysis

	**Countries**	Studies*	**Mean study size**	**Median year of initiation of cART (range)**	**Studies in which frequent viral load monitoring was done (>2 viral loads per year)**	**Studies in which genotypic resistance testing done at viral load <1000 copies per mL**	**Studies in which baseline resistance testing was done**	**Rural clinics**
Eastern Africa (n=143)	3	7	24	2011 (2005–12)	0	1 (14%)	1 (14%)	2 (29%)
Asia (n=356)	4	5	71	2010 (2005–13)	2 (40%)	2 (40%)	2 (40%)	1 (20%)
Eastern Africa (n=143)	3	7	24	2011 (2005–12)	0	1 (14%)	1 (14%)	2 (29%)
Latin America (n=68)	5	6	11	2008 (2000–15)	4 (67%)	2 (67%)	2 (67%)	0 (100%)
North America (n=94)	2	3	47	2008 (2000–14)	3 (100%)	3 (100%)	3 (100%)	3 (100%)
Southern Africa (n=404)	6	15	45	2010 (2005–12)	4 (27%)	4 (27%)	4 (27%)	5 (33%)
West and central Africa (n=107)	5	10	12	2008 (2005–13)	1 (10%)	0	0	0
Western Europe (n=754)	11	20	69	2008 (1998–2013)	20 (100%)	20 (100%)	20 (100%)	0
All (n=1926)	36	66	29	2008 (1998–2015)	34 (52%)	32 (49%)	32 (49%)	11 (17%)

Data are n, range, or n (%). cART=combination antiretroviral therapy.

* Multinational studies were treated as separate studies within each country.

**Table 2:** Participant characteristics and details of antiretroviral therapy

	**Men**	**Age (years)**	**Efavirenz**	**Emtricitabine**	**Baseline CD4 cell count (× 10**^6^**cells per μL)**	**Pre-treatment log**_10_**baseline viral load**	**Number of months on TDF**
Asia (n=356)	229 (67%)	35 (30–39)	300 (84%)	73 (21%)	100 (45–229)	5·00 (4·55–5·68)	14 (9–21)
Eastern Africa (n=143)	57 (40%)	36 (29–44)	56 (39%)	53 (37%)	104 (42–210)	5·58 (5·30–5·83)	14 (12–26)
Latin America (n=68)	19 (70%)	34 (26–44)	65 (96%)	44 (65%)	44 (14–86)	5·47 (5·00–5·93)	26 (11–57)
North America (n=94)	78 (84%)	41 (35–48)	81 (87%)	61 (66%)	144 (25–303)	5·00 (4·59–5·53)	11 (6–24)
Southern Africa (n=404)	147 (36%)	34 (28–40)	290 (72%)	89 (22%)	98 (40–169)	4·80 (3·81–5·47)	18 (12–28)
West and central Africa (n=107)	45 (42%)	36 (30–42)	39 (36%)	79 (74%)	89 (37–166)	5·32 (4·92–5·81)	13 (11–18)
Western Europe (n=754)	571 (76%)	38 (32–44)	653 (87%)	633 (84%)	199 (91–300)	5·00 (4·28–5·46)	12 (7–26)
All (n=1926)	1146 (62%)	37 (30–44)	1485 (77%)	1032 (54%)	139 (53–250)	5·06 (4·45–5·56)	14 (9–27)

Data are n (%) or median (IQR). TDF=tenofovir disoproxil fumarate.
