# Corrections

**DOI:** 10.1016/S1473-3099(16)30089-5

**Published:** 2016-06

*The TenoRes Study Group. Global epidemiology of drug resistance after failure of WHO recommended first-line regimens for adult HIV-1 infection: a multicentre retrospective cohort study.* Lancet Infect Dis *2016; **16:** 565–75*—In this Article, the affiliation for Rami Kantor was incorrect. This correction has been made to the online version as of May 23, 2016.

