# Supplementary material for: Global epidemiology of drug resistance after failure of WHO recommended first-line regimens for adult HIV-1 infection: a multicentre retrospective cohort study
Source: Lancet Infect Dis. 2016 May;16(5):565–75. doi: 10.1016/S1473-3099(15)00536-8 (PMC4835583; doi:10.1016/S1473-3099(15)00536-8)
Supplement: Supplementary appendix [file mmc1.pdf]

THE LANCET Infectious Diseases

Supplementary webappendix

This webappendix formed part of the original submission and has been peer reviewed. We post it as supplied by the authors.

Supplement to: The TenoRes Study Group. Global epidemiology of drug resistance after failure of WHO recommended first-line regimens for adult HIV-1 infection: a multicentre retrospective cohort study. *Lancet Infect Dis* 2016; published online Jan 28. http://dx.doi.org/10.1016/S1473-3099(15)00536-8.

	Country	Income region	Study type	Underlying cohort exclusively first line treated?	Follow-up Active* or passive	N	TDF resistance	VL threshold for genotype	Use of FTC	Use of NVP	Baseline CD4 <100	Baseline viral load >100,000
**Sub Saharan Africa**												
**ACTION**	Nigeria	LMIC	Cohort	Yes	Passive	17	10	1000	17 (100%)	7 (41%)	10 (59%)	-
**ACTION Plus UP**	Nigeria	LMIC	Cohort	Yes	Passive	21	17	1000	18 (86%)	12 (57%)	8 (38%)	-
**Doris Duke Study**	Nigeria	LMIC	Trial	Yes	Active	13	8	1000	0 (0%)	3 (23%)	5 (38%)	7 (54%)
**Harvard/APIN PEPFAR**	Nigeria	LMIC	Cohort	No	Active	20	15	2000	18 (90%)	19 (95%)	16 (80%)	17 (85%)
**CDC Nigeria ADR**	Nigeria	LMIC	Cohort	Yes	Passive	6	3	1000	5 (83%)	6 (100%)	2 (33%)	4 (67%)
**ANRS West Africa**	Senegal, Burkina Faso	LIC	Trial	Yes	Passive	9	3	1000	0 (0%)	2 (22%)	-	-
**DAYANA**	Senegal, Cameroon	LMIC	Trial	Yes	Active	4	0	50	4 (100%)	2 (50%)	1 (25%)	4 (100%)
**Lubumbashi,**	DRC	LIC	Trial	Yes	Active	12	6	1000	12 (100%)	12 (100%)	7 (58%)	9 (75%)
**UVRI/MoH Uganda surveillance study**	Uganda	LIC	Cohort	Yes	Passive	35	19	1000	18 (51%)	24 (69%)	18 (51%)	29 (83%)
**CDC Uganda ADR**	Uganda	LIC	Cohort	Yes	Passive	5	3	1000	4 (80%)	3 (60%)	2 (40%)	-
**CDC/MoH, Tanzania**	Tanzania	LIC	Cohort	No	Active	15	3	1000	12 (80%)	1 (7%)	-	-
**CDC Kenya ADR**	Kenya	LMIC	Cohort	Yes	Passive	43	31	1000	1 (2%)	27 (63%)	17 (40%)	-
**TDF AMPATH**	Kenya	LMIC	Cohort	Yes	Active	27	19	1000	0 (0%)	23 (85%)	-	-
**PASER**	Nigeria, Uganda, South Africa, Kenya, Zambia, Zimbabwe	LMIC	Cohort	No	Active	53	19	1000	52 (98%)	17 (32%)	27 (51%)	35 (66%)
**Aurum, KZN**	South Africa	HMIC	Cohort	No	Active	11	0	1000	9 (82%)	3 (27%)	1 (9%)	0 (0%)
**Africa Centre, KZN**	South Africa	HMIC	Cohort	No	Passive	64	45	1000	0 (0%)	10 (16%)	32 (50%)	-
**Bloemfontein,**	South Africa	HMIC	Cohort	No	Passive	78	59	1000	2 (3%)	16 (21%)	14 (18%)	1 (1%)
**RFVF, Durban**	South Africa	HMIC	Cohort	Yes	Passive	51	34	1000	0 (0%)	7 (14%)	26 (51%)	0 (0%)
**CDC/NCID, KZN,**	South Africa	HMIC	Cohort	Yes		98	49	1000	0 (0%)	33 (34%)	-	-
**Swaziland cohort**	Swaziland	LMIC	Cohort	No	Active	22	12	1000	0 (0%)	5 (23%)	10 (45%)	6 (27%)
**CDC Zambia ADR**	Zambia	LMIC	Cohort	No	Passive	14	8	1000	13 (93%)	1 (7%)	4 (29%)	-
**OCTANE**	Kenya, Botswana,	LMIC	Trial	Yes	Active	36	7	2000	36 (100%)	36 (100%)	16 (44%)	27 (75%)

	Malawi, South Africa, Zambia, Zimbabwe											
**Western Europe and North America**												
**Bichat cohort**	France	HIC	Cohort	No	Passive	30	1	50	30 (100%)	2 (7%)	7 (23%)	13 (43%)
**UK HIV Drug Resistance Database / UK CHIC[1]**	UK	HIC	Cohort	No	Active	155	19	100-500	136 (88%)	0 (0%)	39 (25%)	81 (52%)
**Swiss HIV Cohort Study**	Switzerland	HIC	Cohort	No	Active	13	8	100-500	3 (23%)	2 (15%)	5 (38%)	7 (54%)
**Portuguese HIV-1 DRD**	Portugal	HIC	Cohort	No	Passive	98	32	100-500	79 (81%)	15 (15%)	-	-
**ATRES HCP,**	Spain	HIC	Cohort	Yes	Active	8	1	400	8 (100%)	0 (0%)	-	-
**CORIS**	Spain	HIC	Cohort	No	Passive	62	15	500-1000	54 (87%)	8 (13%)	22 (35%)	37 (60%)
**ATHENA,**	Netherlands	HIC	Cohort	No	Active	58	22	400	39 (67%)	29 (50%)	22 (38%)	36 (62%)
**EU Resist**	Luxembourg, Germany, Sweden, Italy	HIC	Cohort	No	Active	155	20	100-500	132 (85%)	25 (16%)	28 (18%)	56 (36%)
**InfCareSweden**	Sweden	HIC	Cohort	No	Active	50	3	100-500	45 (90%)	0 (0%)	7 (14%)	4 (8%)
**Lazio/Reggio Emilia cohorts**	Italy	HIC	Cohort	No	Active	66	16	100-500	54 (82%)	7 (11%)	18 (27%)	28 (42%)
**ClinSurv, Germany**	Germany	HIC	Cohort	No	Active	53	8	100-500	52 (98%)	13 (25%)	11 (21%)	19 (36%)
**Stanford resistance database**	USA	HIC	Cohort	No	Active	54	14	100-500	48 (89%)	12 (22%)	25 (46%)	29 (54%)
**ATRES GS-903**	USA, UK, Brazil, Germany	HIC	Trial	Yes	Active	34	8	400	0 (0%)	0 (0%)	14 (41%)	15 (44%)
**ATRES GS-934 study**	USA, UK	HIC	Trial	Yes	Active	8	0	400	8 (100%)	0 (0%)	3 (38%)	6 (75%)
**ATRES Vancouver**	Canada	HIC	Cohort	Yes	Active	12	4	400	6 (50%)	0 (0%)	2 (17%)	8 (67%)
**Latin America**												
**Mexican resistance database**	Mexico	HMIC	Cohort	No	Passive	26	9	1000	26 (100%)	2 (8%)	14 (54%)	21 (81%)
**ATRES Guadalajara.**	Mexico	HMIC	Cohort	Yes	Passive	7	1	400	7 (100%)	0 (0%)	5 (71%)	1 (14%)
**Rio de Janeiro**	Brazil	HMIC	Cohort	Yes	Active	12	6	1000	1 (8%)	1 (8%)	-	-
**Nicaragua / Honduras**		LMIC	Cohort	No	Passive	15	5	1000	10 (67%)	0 (0%)	-	-

**Asia**												
**TASER**	Hong Kong, Singapore, Thailand	HMIC	Cohort	No	Active	9	2	1000	5 (56%)	3 (33%)	5 (56%)	3 (33%)
**Thailand national database**	Thailand	HMIC	Cohort	No	Passive	289	118	1000	48 (17%)	44 (15%)	-	-
**National HIV Reference Laboratory**	Israel	HIC	Cohort	No	Passive	12	9	1000	8 (67%)	0 (0%)	4 (33%)	3 (25%)
**YRGCare**	India	LMIC	Cohort	No	Passive	46	10	1000	12 (26%)	9 (20%)	22 (48%)	10 (22%)

[1]only limited UK data (tests between 6-18 months) were included; baseline refers to the pre-cART; * active follow up refers to use of telephone, text message or home visit where patient defaults.

Table S1: Selected characteristics of contributing studies

	Platform used for resistance testing?	Resistance tests external quality assured?	Viral load assays external quality assured?	Number of VL tests per year in cohort.	No of clinic visits per year (nurse or doctor)	Adherence intervention/ counseling at planned clinic visits? (yes/no)	Adherence quantified or measured objectively?	Overall adherence estimate in study if measured formally.
				**Sub Saharan Africa**				
**ACTION, Nigeria**	In-House	Yes	Yes	1	4	Yes	No	NA
**ACTION Plus UP, Nigeria**	In-House	Yes	Yes	1	4	Yes	No	NA
**Doris Duke Study, Nigeria**	In-House	Yes	Yes	4	6	Yes	Pharmacy refill	85% with refill of >95%
**Harvard/APIN PEPFAR Nigeria**	Commercial	Yes	Yes	0	2-3	Yes	No	NA
**CDC Nigeria ADR**	Commercial	Yes	Yes	0	4	Yes	No	NA
**ANRS West Africa**	Commercial	Yes	Yes	2	2	Yes	No	NA
**Lubumbashi, DRC**	Commercial	Yes	Yes	2	4	Yes	Pill counts	60% with >95% adherence
**DAYANA, west/central Africa**	Commercial	Yes	Yes	6	6	Yes	No	NA
**UVRI/MoH Uganda surveillance study**	In-House	Yes	Yes	0	4	Yes	No	NA
**CDC Uganda ADR**	Commercial	Yes	Yes	0	4	Yes	No	NA
**CDC/MoH, Tanzania**	Commercial	Yes	Yes	0	4	Yes	No	NA
**CDC Kenya ADR**	Commercial	Yes	Yes	0	4	Yes	No	NA
**TDF AMPATH, Kenya**	In-House	Yes	Yes	0	12	Yes	No	NA
**PASER-M, multiple sites**	In-House	Yes	Yes	Variable	4	Yes	No	NA
**Aurum, South Africa**	In-House	Yes	Yes	2	4	Yes	No	NA
**Africa Centre, South Africa**	In-House	Yes	Yes	2	12	Yes	No	NA
**Bloemfontein, South Africa**	In-House	Yes	Yes	2	2	Yes	No	NA
**RFVF, South Africa**	In-House	Yes	Yes	2	2	Yes	Pill count adherence ratio (PCAR)	90% with >90% PCAR
**CDC / NCID South Africa**	In-House	Yes	Yes	0	6			

**Swaziland cohort**	In-House	Yes	Yes	0	4-6	Yes	No	NA
**CDC Zambia ADR**	Commercial	Yes	Yes	0	4	Yes	No	NA
**OCTANE, multiple sites**	Commercial	Yes	Yes	4	4	Yes	Pill counts	NA
				**Western Europe and North America**				
**Bichat cohort, Paris France**	In-House	Yes	Yes	4	4	Yes	No	NA
**UK HIV Drug Resistance Database/ UK CHIC**[1]	In-House and Commercial	Yes	Yes	2-4	2-4	Yes	No	NA
**Swiss HIV Cohort Study**	In-House and Commercial	Yes	Yes	3-4	3-4	Yes	No	NA
**Portuguese Resistance Database**[1]	Commercial	Yes	Yes	1-4	1-4	Yes	No	NA
**ATRES HCP, Spain**	In-House and Commercial	Yes	Yes	3-4	3-4	Yes	No	NA
**CoRIS, Spain**	In-House and Commercial	Yes	Yes	3-4	3-4	Yes	No	NA
**ATHENA, Netherlands**	Commercial	Yes	Yes	2	3	Yes	No	NA
**EU Resist, multiple sites**	In-House 40%, Commercial 60%	Yes	Yes	4	4	Yes	No	NA
**InfCare, Sweden**	Commercial	Yes	Yes	3-4	2-4	Yes	No	NA
**Lazio/Reggio Emilia cohorts, Italy**	In-House and Commercial	Yes	Yes	3-4	3-4	Yes	No	NA
**ClinSurv, Germany**	In-House and Commercial	Yes	Yes	4	4	Yes	No	NA
**Stanford resistance database, USA**	In-House	Yes	Yes	4	4	Yes	No	NA
**GS-903, USA, UK**	Commercial	Yes	Yes	14	14	Yes	No	NA
**GS-934, USA, UK**	Commercial	Yes	Yes	8	8	Yes	Yes	NA
**ATRES Vancouver, Canada**	In-House	Yes	Yes	3-4	3-4	Yes	No	NA
				**Latin America**				
**Rio de Janeiro, Brazil**	In-House and Commercial	Yes	Yes	3-4	3-4	Yes	No	NA
**Mexican resistance database**	In-House	Yes	Yes	3	3	Yes	No	NA
**Nicaragua / Honduras**	In-House	Yes	Yes	2	2	Yes	No	NA
**ATRES, Mexico**	In-House	Yes	Yes	3	3	Yes	No	NA
				**Asia**				
**TASER**	In-House	Yes	Yes	2	2	Yes	No	NA
**Thailand national drug resistance**	In-House	Yes	Yes	1	2	Yes	No	NA

**National HIV Reference Laboratory, Israel**	Commercial	Yes	Yes	2	2	Yes	No	NA
**YRGCare, India**	In-House	Yes	Yes	1	2	Yes	No	NA

Table S2: Quality indicators of contributing datasets

		Nevirapine versus efavirenz		Lamivudine versus emtricitabine		Baseline CD4 (cells/mm3)		Baseline viral load (copies/ml)	
		N	Odds Ratio (95% CI)	N	Odds Ratio (95% CI)	N	Odds Ratio (95% CI)	N	Odds Ratio (95% CI)
**NNRTI**	EFV	-	-	1053	1.79 (1.38 to 2.32)	866	1.89 (1.56 to 2.31)	702	1.35 (1.02 to 1.79)
	NVP	-	-	175	0.96 (0.71 to 1.31)	246	1.25 (1.00 to 1.55)	181	1.15 (0.83 to 1.61)
**Cytosine analogue**	FTC	619	1.88 (1.45 to 2.44)		-	736	1.81 (1.44 to 2.29)	698	1.38 (1.04 to 1.84)
	3TC	779	1.35 (1.19 to 1.55)		-	353	1.40 (1.16 to 1.69)	175	1.16 (0.82 to 1.63)
**Baseline CD4 (cells/mm3)**	>100	439	2.15 (1.61 to 2.87)	491	1.59 (1.10 to 2.31)	-	-	505	1.27 (0.90 to 1.79)
	>100	309	1.31 (1.02 to 1.69)	188	1.09 (0.73 to 1.63)	-	-	265	0.94 (0.71 to 1.23)
**Baseline viral load (copies/ml)**	<100,000	232	2.18 (1.46 to 3.26)	297	2.19 (1.41 to 3.40)	320	1.81 (1.29 to 2.54)		-
	>100,000	292	1.79 (1.27 to 2.52)	304	1.03 (0.72 to 1.49)	437	1.57 (1.19 to 2.07)		-
**Country-level income category**	Other	951	1.41 (1.24 to 1.60)	603	1.43 (1.06 to 1.93)	481	1.52 (1.29 to 1.80)	248	1.39 (1.00 to 1.94)
	HMIC	575	2.10 (1.54 to 2.86)	774	1.60 (1.21 to 2.12)	659	1.80 (1.34 to 2.42)	666	1.21 (0.91 to 1.62)
**Study design**	Cohort	1513	1.55 (1.37 to 1.74)	1377	1.51 (1.23 to 1.85)	1058	1.57 (1.35 to 1.82)	832	1.28 (1.01 to 1.61)
	Trial	13	1.11 (0.43 to 2.86)	Insufficient data		82	2.29 (1.23 to 4.28)	82	1.28 (0.69 to 2.38)
**Type of follow up**	Passive	899	1.45 (1.25 to 1.67)	740	1.30 (1.00 to 1.69)	436	1.48 (1.25 to 1.75)	204	1.52 (1.00 to 2.32)
	Active	529	1.79 (1.39 to 2.31)	637	1.98 (1.41 to 2.78)	704	1.81 (1.40 to 2.35)	710	1.20 (0.93 to 1.55)

Table S3: Sub group analysis of factors associated with tenofovir resistance.

N: number available for each analysis

	Cytosine analogue resistance		Major NNRTI resistance	
	Number in analysis	Odds Ratio (95% CI)	Number in analysis	Odds Ratio (95% CI)
**Baseline CD4 <100 cells/mm^3(vs >100)**	1137	1.35 (1.20 to 1.51)	1062	1.17 (1.08 to 1.27)
**Baseline viral load >100,000 copies HIV-1 RNA/ ml (vs <100,000)**	918	1.13 (0.97 to 1.32)	857	1.05 (0.94 to 1.17)
**Nevirapine (vs efavirenz)**	1542	1.28 (1.17 to 1.40)	1347	1.15 (1.08 to 1.22)
**Lamivudine (vs emtricitabine)**	1378	1.24 (1.04 to 1.47)	1346	1.21 (1.10 to 1.33)

Table S4: Odds ratios for pre therapy CD4 count/viral load and co-administered drugs on resistance to cytosine analogue and NNRTI following viral failure.

	Random effects meta analysis (primary analysis)		Fixed effects meta analysis		Multi-level logistic regression adjusted for study region		Fully adjusted multi-level logistic regression*	
	N	95% CI	N	95% CI	N	95% CI	N	95% CI
NVP (vs EFV)	1526	1.46 (1.28 to 1.67)	1526	1.54 (1.36 to 1.73)	1926	2.34 (1.78 to 3.07)	933	2.94 (1.92 to 4.52)
3TC (vs FTC)	1377	1.48 (1.20 to 1.82)	1377	1.51 (1.23 to 1.85)	1926	2.33 (1.74 to 3.11)	933	2.37 (1.57 to 3.58)
Baseline CD4 <100 (vs >100)	1140	1.50 (1.27 to 1.77)	1140	1.61 (1.39 to 1.86)	1191	2.75 (2.06 to 3.69)	933	2.70 (1.90 to 3.85)
Baseline viral load >100,000 (vs <100,000)	914	1.17 (0.94 to 1.44)	914	1.28 (1.03 to 1.59)	974	1.52 (1.08 to 2.13)	933	1.32 (0.91 to 1.91)

*Fully adjusted model is adjusted for subtype C, NVP vs EFV, 3TC vs FTC, baseline CD4 and baseline viral load

Table S5: **Odds ratios for covariates and tenofovir resistance in by choice of method used for analysis**

Study	Number of participants contributing to analysis					
	TDF resistance	Baseline CD4	Baseline viral load	FTC vs EFV	NVP vs EFV	Adjusted regression model
**All studies**	**1926**	**1140**	**914**	**1377**	**1526**	**933**
**Asia**	**356**	**60**	**32**	**355**	**343**	**31**
China	1	0	0	0	0	1
TASER	1	0	0	0	0	1
India	46	46	17	46	46	17
YRGCare	46	46	17	46	46	17
Israel	12	6	7	12	0	5
National HIV Reference Laboratory	12	6	7	12	0	5
Thailand	297	8	8	297	297	8
TASER	8	8	8	8	8	8
Thailand national database	289	0	0	289	289	0
**Eastern Africa**	**143**	**93**	**49**	**98**	**139**	**53**
Kenya	74	40	0	43	70	4
CDC Kenya ADR	43	40	0	43	43	0
OCTANE	4	0	0	0	0	4
TDF AMPATH	27	0	0	0	27	0
Tanzania, United Republic of	15	0	0	15	15	0
CDC/MoH, Tanzania	15	0	0	15	15	0
Uganda	54	53	49	40	54	49
CDC Uganda ADR	5	4	0	5	5	0
PASER	14	14	14	0	14	14
UVRI/MoH Uganda surveillance study	35	35	35	35	35	35
**Latin America**	**68**	**30**	**38**	**23**	**38**	**29**
Argentina	2	2	2	0	0	2
ATRES GS-903	2	2	2	0	0	2
Brazil	18	6	6	12	12	6
ATRES GS-903	6	6	6	0	0	6
Rio de Janeiro	12	0	0	12	12	0
Honduras	11	0	0	11	0	0
Nicaragua / Honduras	11	0	0	11	0	0
Mexico	33	22	30	0	26	21
ATRES Guadalajara	7	6	5	0	0	5
Mexican national resistance database	26	16	25	0	26	16
Nicaragua	4	0	0	0	0	0
Nicaragua / Honduras	4	0	0	0	0	0
**North America**	**94**	**85**	**82**	**66**	**54**	**89**
Canada	12	12	12	12	0	12
ATRES Vancouver	12	12	12	12	0	12
United States	82	73	70	54	54	77
ATRES GS-903	21	21	21	0	0	21
ATRES GS-934	7	0	0	0	0	7

Stanford	54	52	49	54	54	49
**Southern Africa**	**404**	**216**	**83**	**92**	**360**	**103**
Botswana	3	0	0	0	0	3
OCTANE	3	0	0	0	0	3
Malawi	7	7	7	0	0	7
OCTANE	7	7	7	0	0	7
South Africa	350	168	50	78	324	65
Africa Centre	64	55	0	0	64	0
Aurum	11	0	0	0	0	10
Bloemfontein	78	21	3	78	78	1
RFVF, Durban	51	45	0	0	51	8
CDC South Africa	98	0	0	0	98	0
OCTANE	15	15	15	0	0	15
PASER	11	10	11	0	11	10
Swaziland						
Swaziland	22	22	21	0	22	21
Zambia	37	35	20	14	36	21
CDC Zambia ADR	14	13	0	14	14	0
OCTANE	1	0	0	0	0	1
PASER	22	22	20	0	22	20
Zimbabwe	7	6	6	0	0	7
OCTANE	6	6	6	0	0	6
PASER	1	0	0	0	0	1
**West/Central Africa**	107	90	55	47	71	58
Burkina Faso	3	0	0	0	0	0
ANRS West Africa, Burkina Faso	3	0	0	0	0	0
Cameroon	3	0	0	0	0	3
DAYANA, Cameroun	3	0	0	0	0	3
DRC	12	12	12	0	0	12
Lubumbashi	12	12	12	0	0	12
Nigeria	82	78	43	47	71	42
ACTION, Nigeria	17	15	0	0	17	0
ACTION Plus UP, Nigeria	21	20	0	21	21	0
CDC Nigeria ADR	6	5	5	6	0	4
Doris Duke study	13	13	13	0	13	13
Harvard/APIN PEPFAR	20	20	20	20	20	20
PASER	5	5	5	0	0	5
Senegal	7	0	0	0	0	1
ANRS West Africa	6	0	0	0	0	0
DAYANA	1	0	0	0	0	1
**Western Europe**	**754**	**566**	**575**	**696**	**521**	**570**
Belgium	9	9	9	9	9	9
EU Resist	9	9	9	9	9	9
France	31	28	27	0	30	27
ATRES GS-903	1	0	0	0	0	1
Bichat cohort	30	28	27	0	30	26

Germany	68	43	42	53	53	57
ATRES GS-903	1	0	0	0	0	1
ClinServ	53	43	42	53	53	42
EU Resist	14	0	0	0	0	14
Italy	108	99	99	106	106	101
ATRES GS-903	2	0	0	0	0	2
EU Resist	40	39	39	40	40	39
Lazio/Reggio Emilia cohorts, Italy	66	60	60	66	66	60
Luxembourg	14	13	14	14	14	13
EU Resist	14	13	14	14	14	13
Netherlands	58	57	57	58	58	56
ATHENA, Netherlands	58	57	57	58	58	56
Portugal	150	29	47	150	150	29
EU Resist	52	29	47	52	52	29
Portuguese HIV-1 DRD	98	0	0	98	98	0
Spain	70	61	60	62	62	60
ATRES HCP	8	0	0	0	0	0
CORIS	62	61	60	62	62	60
Sweden	76	76	73	76	26	73
EU Resist	26	26	23	26	26	23
InfCare	50	50	50	50	0	50
Switzerland	13	13	13	13	13	13
Swiss HIV Cohort Study	13	13	13	13	13	13
United Kingdom	157	138	134	155	0	132
ATRES GS-903	1	0	0	0	0	1
ATRES GS-934	1	0	0	0	0	1
UK HIV Drug Resistance Database / UK CHIC	155	138	134	155	0	130

Table S6: Numbers of participants from each study, country and region contributing to specific analyses.

Figure S1: Pooled odds ratios for tenofovir resistance following viral failure for baseline A) viral load ≥100,000 vs. <100,000 copies HIV-1 RNA/ml B) lamivudine (3TC) vs emtricitabine (FTC) C) nevirapine (NVP) vs efavirenz (EFV)

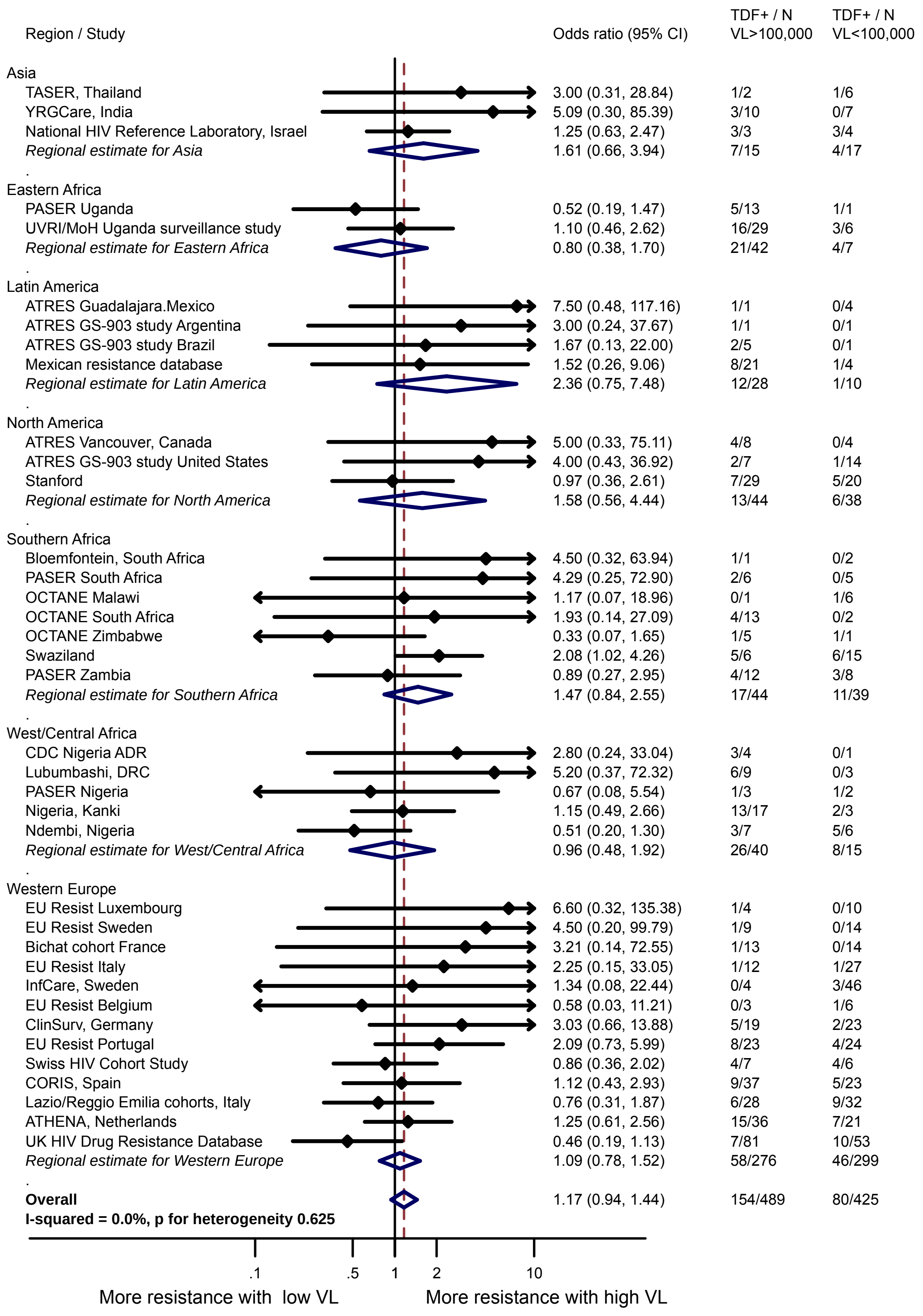

Odds ratio for TDF resistance with baseline viral
[illegible]

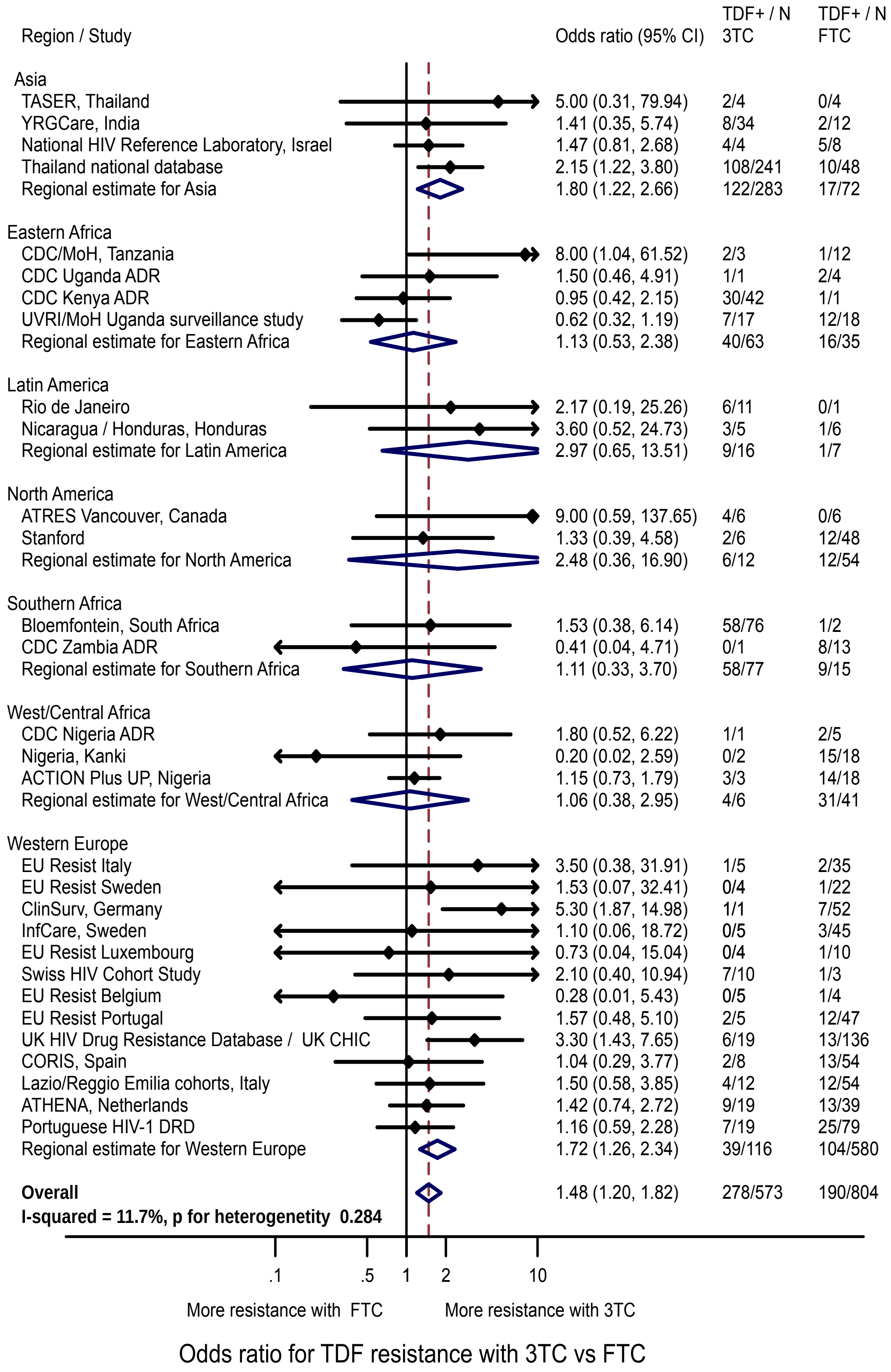
Region / Study
Odds ratio (95% CI)
TDF+ / N 3TC
TDF+ / N FTC
Asia
TASER, Thailand 5.00 (0.31, 79.94) 2/4 0/4
YRGCare, India 1.41 (0.35, 5.74) 8/34 2/12
National HIV Reference Laboratory, Israel 1.47 (0.81, 2.68) 4/4 5/8
Thailand national database 2.15 (1.22, 3.80) 108/241 10/48
Regional estimate for Asia 1.80 (1.22, 2.66) 122/283 17/72
Eastern Africa
CDC/MoH, Tanzania 8.00 (1.04, 61.52) 2/3 1/12
CDC Uganda ADR 1.50 (0.46, 4.91) 1/1 2/4
CDC Kenya ADR 0.95 (0.42, 2.15) 30/42 1/1
UVRI/MoH Uganda surveillance study 0.62 (0.32, 1.19) 7/17 12/18
Regional estimate for Eastern Africa 1.13 (0.53, 2.38) 40/63 16/35
Latin America
Rio de Janeiro 2.17 (0.19, 25.26) 6/11 0/1
Nicaragua / Honduras, Honduras 3.60 (0.52, 24.73) 3/5 1/6
Regional estimate for Latin America 2.97 (0.65, 13.51) 9/16 1/7
North America
ATRES Vancouver, Canada 9.00 (0.59, 137.65) 4/6 0/6
Stanford 1.33 (0.39, 4.58) 2/6 12/48
Regional estimate for North America 2.48 (0.36, 16.90) 6/12 12/54
Southern Africa
Bloemfontein, South Africa 1.53 (0.38, 6.14) 58/76 1/2
CDC Zambia ADR 0.41 (0.04, 4.71) 0/1 8/13
Regional estimate for Southern Africa 1.11 (0.33, 3.70) 58/77 9/15
West/Central Africa
CDC Nigeria ADR 1.80 (0.52, 6.22) 1/1 2/5
Nigeria, Kanki 0.20 (0.02, 2.59) 0/2 15/18
ACTION Plus UP, Nigeria 1.15 (0.73, 1.79) 3/3 14/18
Regional estimate for West/Central Africa 1.06 (0.38, 2.95) 4/6 31/41
Western Europe
EU Resist Italy 3.50 (0.38, 31.91) 1/5 2/35
EU Resist Sweden 1.53 (0.07, 32.41) 0/4 1/22
ClinSurv, Germany 5.30 (1.87, 14.98) 1/1 7/52
InfCare, Sweden 1.10 (0.06, 18.72) 0/5 3/45
EU Resist Luxembourg 0.73 (0.04, 15.04) 0/4 1/10
Swiss HIV Cohort Study 2.10 (0.40, 10.94) 7/10 1/3
EU Resist Belgium 0.28 (0.01, 5.43) 0/5 1/4
EU Resist Portugal 1.57 (0.48, 5.10) 2/5 12/47
UK HIV Drug Resistance Database / UK CHIC 3.30 (1.43, 7.65) 6/19 13/136
CORIS, Spain 1.04 (0.29, 3.77) 2/8 13/54
Lazio/Reggio Emilia cohorts, Italy 1.50 (0.58, 3.85) 4/12 12/54
ATHENA, Netherlands 1.42 (0.74, 2.72) 9/19 13/39
Portuguese HIV-1 DRD 1.16 (0.59, 2.28) 7/19 25/79
Regional estimate for Western Europe 1.72 (1.26, 2.34) 39/116 104/580
Overall 1.48 (1.20, 1.82) 278/573 190/804
I-squared = 11.7%, p for heterogenetity 0.284
.1
.5
1
2
10
More resistance with FTC
More resistance with 3TC
Odds ratio for TDF resistance with 3TC vs FTC

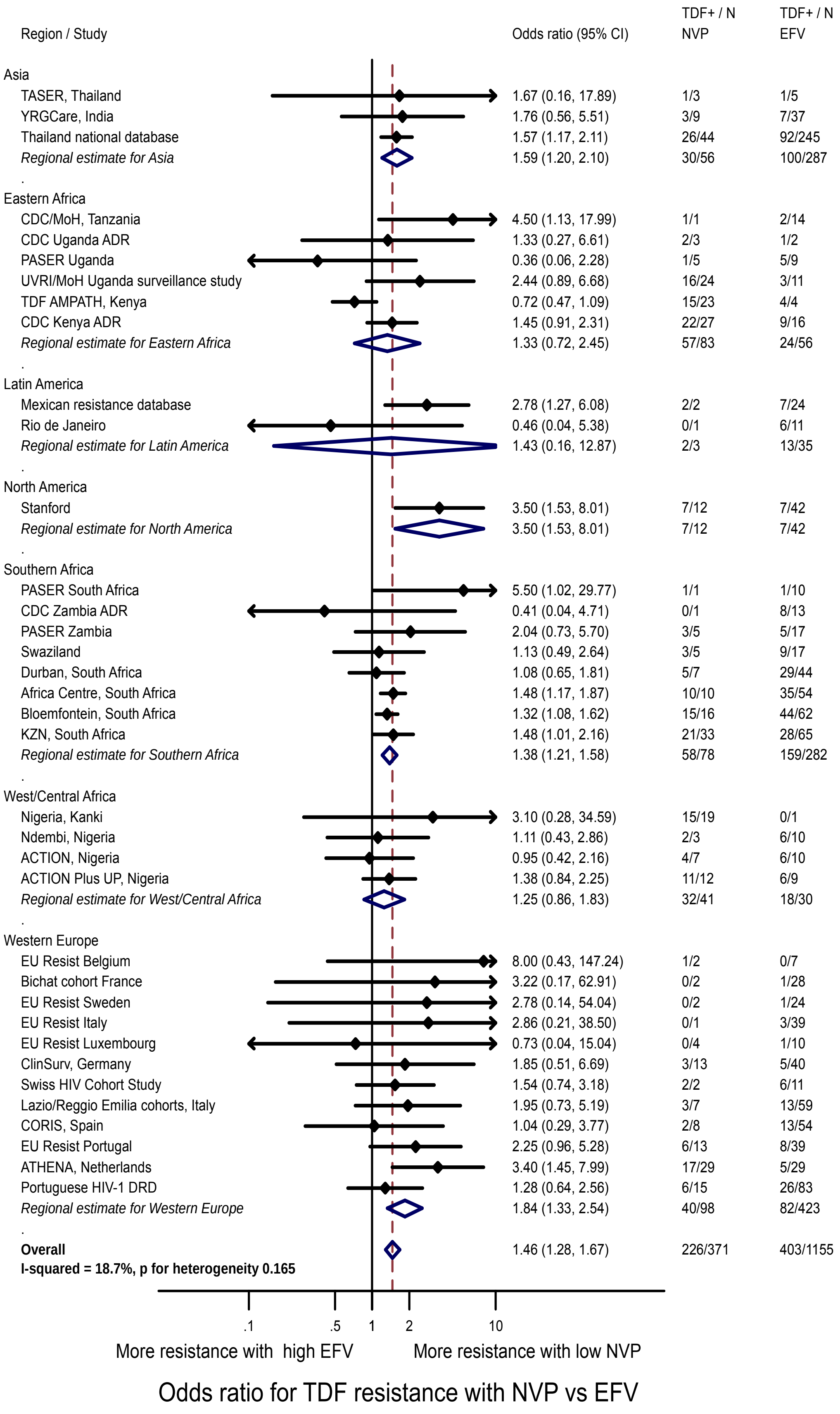

Region / Study
Odds ratio (95% CI)
TDF+ / N NVP
TDF+ / N EFV
Asia
TASER, Thailand 1.67 (0.16, 17.89) 1/3 1/5
YRGCare, India 1.76 (0.56, 5.51) 3/9 7/37
Thailand national database 1.57 (1.17, 2.11) 26/44 92/245
Regional estimate for Asia 1.59 (1.20, 2.10) 30/56 100/287
Eastern Africa
CDC/MoH, Tanzania 4.50 (1.13, 17.99) 1/1 2/14
CDC Uganda ADR 1.33 (0.27, 6.61) 2/3 1/2
PASER Uganda 0.36 (0.06, 2.28) 1/5 5/9
UVRI/MoH Uganda surveillance study 2.44 (0.89, 6.68) 16/24 3/11
TDF AMPATH, Kenya 0.72 (0.47, 1.09) 15/23 4/4
CDC Kenya ADR 1.45 (0.91, 2.31) 22/27 9/16
Regional estimate for Eastern Africa 1.33 (0.72, 2.45) 57/83 24/56
Latin America
Mexican resistance database 2.78 (1.27, 6.08) 2/2 7/24
Rio de Janeiro 0.46 (0.04, 5.38) 0/1 6/11
Regional estimate for Latin America 1.43 (0.16, 12.87) 2/3 13/35
North America
Stanford 3.50 (1.53, 8.01) 7/12 7/42
Regional estimate for North America 3.50 (1.53, 8.01) 7/12 7/42
Southern Africa
PASER South Africa 5.50 (1.02, 29.77) 1/1 1/10
CDC Zambia ADR 0.41 (0.04, 4.71) 0/1 8/13
PASER Zambia 2.04 (0.73, 5.70) 3/5 5/17
Swaziland 1.13 (0.49, 2.64) 3/5 9/17
Durban, South Africa 1.08 (0.65, 1.81) 5/7 29/44
Africa Centre, South Africa 1.48 (1.17, 1.87) 10/10 35/54
Bloemfontein, South Africa 1.32 (1.08, 1.62) 15/16 44/62
KZN, South Africa 1.48 (1.01, 2.16) 21/33 28/65
Regional estimate for Southern Africa 1.38 (1.21, 1.58) 58/78 159/282
West/Central Africa
Nigeria, Kanki 3.10 (0.28, 34.59) 15/19 0/1
Ndembi, Nigeria 1.11 (0.43, 2.86) 2/3 6/10
ACTION, Nigeria 0.95 (0.42, 2.16) 4/7 6/10
ACTION Plus UP, Nigeria 1.38 (0.84, 2.25) 11/12 6/9
Regional estimate for West/Central Africa 1.25 (0.86, 1.83) 32/41 18/30
Western Europe
EU Resist Belgium 8.00 (0.43, 147.24) 1/2 0/7
Bichat cohort France 3.22 (0.17, 62.91) 0/2 1/28
EU Resist Sweden 2.78 (0.14, 54.04) 0/2 1/24
EU Resist Italy 2.86 (0.21, 38.50) 0/1 3/39
EU Resist Luxembourg 0.73 (0.04, 15.04) 0/4 1/10
ClinSurv, Germany 1.85 (0.51, 6.69) 3/13 5/40
Swiss HIV Cohort Study 1.54 (0.74, 3.18) 2/2 6/11
Lazio/Reggio Emilia cohorts, Italy 1.95 (0.73, 5.19) 3/7 13/59
CORIS, Spain 1.04 (0.29, 3.77) 2/8 13/54
EU Resist Portugal 2.25 (0.96, 5.28) 6/13 8/39
ATHENA, Netherlands 3.40 (1.45, 7.99) 17/29 5/29
Portuguese HIV-1 DRD 1.28 (0.64, 2.56) 6/15 26/83
Regional estimate for Western Europe 1.84 (1.33, 2.54) 40/98 82/423
Overall 1.46 (1.28, 1.67) 226/371 403/1155
I-squared = 18.7%, p for heterogeneity 0.165
.1
.5
1
2
10
More resistance with high EFV
More resistance with low NVP
Odds ratio for TDF resistance with NVP vs EFV

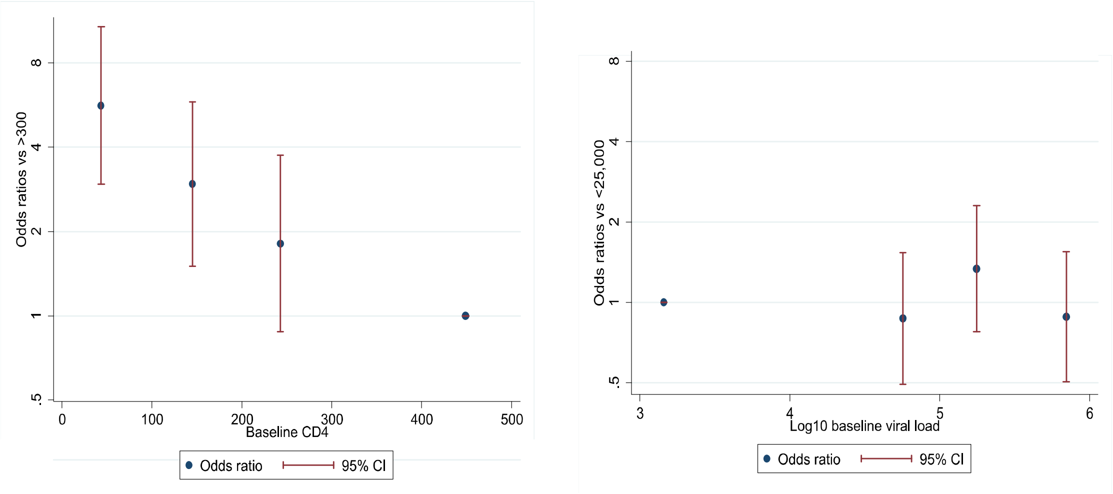

Figure S2: Odds ratios for TDF resistance using fully adjusted multivariate regression models: Left panel for baseline CD4 categories [<100, 100-200, 201-300, >300 (reference category)]; right panel for baseline viral load categories [<25,000 (reference); 25,000-100,000; 100,001-300,000; >300,000].

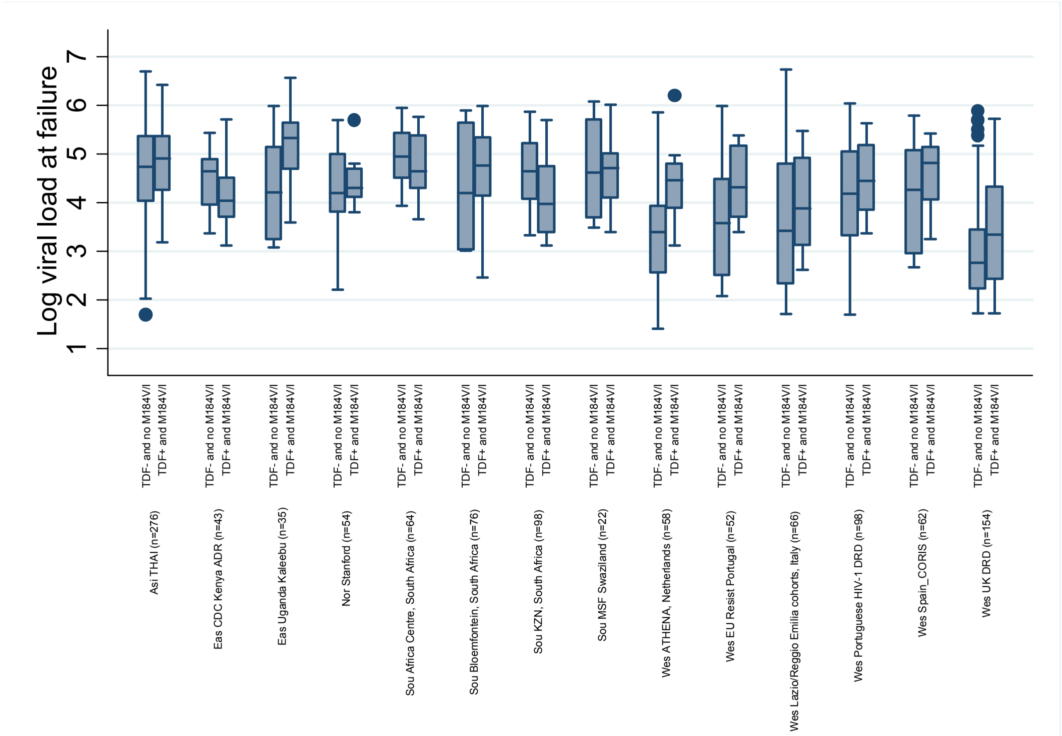

Figure S3: Box plot of log viral load by presence or absence of tenofovir + lamivudine resistance at viral failure in studies with at least 10 patients* with TDF and lamivudine resistance and a viral load measurement at treatment failure.

Supplementary methods

Data were not completely available for all covariates. In particular pre-ART virus load was not available in many LMIC regions that did not to implement routine VL monitoring. In addition, pre-ART resistance tests were also rarely available in LMIC regions. Because transmitted tenofovir resistance has historically been rare in LMICs [1,2], we assumed that any tenofovir resistance following virological failure emerged during first-line tenofovir-containing ART. In high resource settings where baseline resistance testing has been recommended for routinely testing ART naïve patients since 2003[3], clinically significant resistance to tenofovir would have been detected by resistance testing at baseline and alternative NRTI used, thus precluding such individuals from this study.

In some LMICs, it was unclear whether certain individuals had previously received the thymidine analogues zidovudine or stavudine. In these particular cohorts the prevalence of thymidine analogue mutations (TAMs) at the time of virological failure was higher than in cohorts of patients in whom it was certain that previous AZT or d4T had not been used. Based on this finding and the rarity of TAMs in patients receiving tenofovir (because of the known antagonism between TAMs and the key tenofovir mutation K65R[4]) we excluded from our primary analysis the 202 patients who had TAMs at the time of VF based on genotype results.

We systematically assessed the quality of participating studies (supplementary table 2). Variables included availability of adherence support, frequency of clinical follow up, frequency of viral load measurement and where objective measures were available, adherence levels. We also assessed the quality of clinical virology: although both laboratory-developed resistance test assays as well as commercial resistance test kits were used across income groups, all were subject to external quality assurance programs, as were viral load assays (supplementary table 2). No studies were excluded on grounds of quality.

Study groups

Uganda Virus Research Institute/Ministry of Health (UVRI/MoH) Uganda surveillance study: Fred Lyagoba, Tom Lutalo', Anne, Kapaata, Faith Nanyonga, Chris Parry, Norah Namuwenge, Robert Downing

The Cross Sectional Survey of Acquired Drug Resistance Study at Sentinel Sites Study Team:

University of Maryland: Robert Redfield, Sekela Mwakyusa, Peter Memiah, Martine Etienne-Mesubi, Sandra Medina-Moreno, Andrew Kigombola, Sylvia Ojoo, Francesca Odhiambo,

Westat: Karen Megazzini, Annie Lo, Laura Alvarz-Rojas

Kenya: Sarah Masyuko, Shobha Vakil, Ibrahim Mohammed, David Kimanga, Maureen Kimani, and the Kenya National HIVDR working group

Tanzania/Zanzibar: Bonita Kilama, Mathias Abuya, Ben Rabiel, Sophia Mohamed, Jullu Boniphace, Geoffrey Somi, Ahmed Khatibu and the Tanzanian National HIVDR working group

CDC-Kenya: Lucy Nganga, Evelyn Ngugi, Andrea Kim, Jane Mwangi, Anthony Gichangi, Abraham Kitana, Frankline Onchiri, Frederick Miruka

CDC-Tanzania: Mary Kibona, Julius Muhumuza, Jennifer Ward, John Rogers, Duncan Donnay, Rama Mwiru, Godwin Munuo, Mohamed Mfaume, Eunice Mmari, Michelle Roland.

CDC-Atlanta: Tedd Ellerbrock, Laura Broyles, Jennifer Sabatier, Emilia Rivadeneira, Guoqing Zhang, Joshua R. DeVos, Nicolas Wager, Duping Zheng, Karidia Diallo and R. Suzanne Beard.

The ClinSurv Study Group: Gerd Fätkenheuer, Eugen Schülter, Hans-Jürgen Stellbrink, Christian Noah, Björn-Erik Ole Jensen, Matthias Stoll, Johannes R. Bogner, Josef Eberle, Karolin Meixenberger, Claudia Kücherer, Daniel Schmidt, Christian Kollan, Osamah Hamouda, Barbara Bartmeyer

ACTG 5208 study team: Shahin Lockman, John Mellors, Michael Hughes, Fred Sawe, James McIntyre, Judy Currier.

UK HIV Drug Resistance Database

Steering Committee: Celia Aitken (Gartnavel General Hospital, Glasgow); David Asboe, Anton Pozniak (Chelsea & Westminster Hospital, London); Patricia Cane (Public Health England, Porton Down); David Chadwick (South Tees Hospitals NHS Trust, Middlesbrough); Duncan Churchill (Brighton and Sussex University Hospitals NHS Trust); Duncan Clark (St Bartholomew's and The London NHS Trust); Simon Collins (HIV i-Base, London); Valerie Delpech (Centre for Infections, Public Health England); Samuel Douthwaite (Guy's and St. Thomas' NHS Foundation Trust, London); David Dunn, Esther Fearnhill, Kholoud Porter, Anna Tostevin, Ellen White (MRC Clinical Trials Unit at UCL, London); Christophe Fraser (Imperial College London); Anna Maria Geretti (Institute of Infection and Global Health, University of Liverpool); Antony Hale (Leeds Teaching Hospitals NHS Trust); Stéphane Hué (University College London); Steve Kaye (Imperial College, London); Paul Kellam (Wellcome Trust Sanger Institute & University College London Medical School); Linda Lazarus (Expert Advisory Group on AIDS Secretariat, Public Health England); Andrew Leigh-Brown (University of Edinburgh); Tamyo Mbisa (Virus Reference Department, Public Health England); Nicola Mackie (Imperial NHS Trust, London); Samuel Moses (King's College Hospital, London); Chloe Orkin (St. Bartholomew's Hospital, London); Eleni Nastouli, Deenan Pillay, Andrew Phillips, Caroline Sabin (University College London Medical School. London); Erasmus Smit (Public Health England, Birmingham Heartlands Hospital); Kate Templeton (Royal Infirmary of Edinburgh); Peter Tilston (Manchester Royal Infirmary);

Daniel Webster (Royal Free NHS Trust, London); Ian Williams (Mortimer Market Centre, London); Hongyi Zhang (Addenbrooke's Hospital, Cambridge).

Coordinating Centre: MRC Clinical Trials Unit at UCL (Hannah Castro, David Dunn, Keith Fairbrother, Esther Fearnhill, Kholoud Porter, Anna Tostevin, Ellen White)

Centres contributing data: Clinical Microbiology and Public Health Laboratory, Addenbrooke's Hospital, Cambridge (Jane Greatorex); Guy's and St. Thomas' NHS Foundation Trust, London (Siobhan O'Shea, Jane Mullen); PHE – Public Health Laboratory, Birmingham Heartlands Hospital, Birmingham (Erasmus Smit); PHE – Virus Reference Department, London (Tamyo Mbisa); Imperial College Health NHS Trust, London (Alison Cox); King's College Hospital, London (Richard Tandy); Medical Microbiology Laboratory, Leeds Teaching Hospitals NHS Trust (Tracy Fawcett); Specialist Virology Centre, Liverpool (Mark Hopkins, Lynn Ashton); Department of Clinical Virology, Manchester Royal Infirmary, Manchester (Peter Tilston); Department of Virology, Royal Free Hospital, London (Claire Booth, Ana Garcia-Diaz); Edinburgh Specialist Virology Centre, Royal Infirmary of Edinburgh (Jill Shepherd); Department of Infection & Tropical Medicine, Royal Victoria Infirmary, Newcastle (Matthias L Schmid, Brendan Payne); South Tees Hospitals NHS Trust, Middlesbrough (David Chadwick); Department of Virology, St Bartholomew's and The London NHS Trust (Spiro Pereira, Jonathan Hubb); Molecular Diagnostic Unit, Imperial College, London (Steve Kaye); University College London Hospitals (Stuart Kirk); West of Scotland Specialist Virology Laboratory, Gartnavel, Glasgow (Rory Gunson, Amanda Bradley-Stewart, Celia Aitken).

The members of the Swiss HIV Cohort Study are: Aubert V, Battegay M, Bernasconi E, Böni J, Bucher HC, Burton-Jeangros C, Calmy A, Cavassini M, Dollenmaier G, Egger M, Elzi L, Fehr J, Fellay J, Furrer H (Chairman of the Clinical and Laboratory Committee), Fux CA, Gorgievski M, Günthard H (President of the SHCS), Haerry D (deputy of "Positive Council"),

Hasse B, Hirsch HH, Hoffmann M, Hösli I, Kahlert C, Kaiser L, Keiser O, Klimkait T, Kouyos R, Kovari H, Ledergerber B, Martinetti G, Martinez de Tejada B, Metzner K, Müller N, Nadal D, Nicca D, Pantaleo G, Rauch A (Chairman of the Scientific Board), Regenass S, Rickenbach M (Head of Data Center), Rudin C (Chairman of the Mother & Child Substudy), Schöni-Affolter F, Schmid P, Schüpbach J, Speck R, Tarr P, Trkola A, Vernazza P, Weber R, Yerly S.

Athena cohort clinical centres (* *denotes site coordinating physician)*

Academic Medical Centre of the University of Amsterdam: *HIV treating physicians:* J.M. Prins*, T.W. Kuijpers, H.J. Scherpbier, J.T.M. van der Meer, F.W.M.N. Wit, M.H. Godfried, P. Reiss, T. van der Poll, F.J.B. Nellen, S.E. Geerlings, M. van Vugt, D. Pajkrt, J.C. Bos, W.J. Wiersinga, M. van der Valk, A. Goorhuis, J.W. Hovius. *HIV nurse consultants:* J. van Eden, A. Henderiks, A.M.H. van Hes, M. Mutschelknauss, H.E. Nobel, F.J.J. Pijnappel, A.M. Westerman. *HIV clinical virologists/chemists:* S. Jurriaans, N.K.T. Back, H.L. Zaaijer, B. Berkhout, M.T.E. Cornelissen, C.J. Schinkel, X.V. Thomas. **Admiraal De Ruyter Ziekenhuis, Goes:** *HIV treating physicians:* M. van den Berge, A. Stegeman. *HIV nurse consultants:* S. Baas, L. Hage de Looff. *HIV clinical virologists/chemists:* D. Versteeg. **Catharina Ziekenhuis, Eindhoven:** *HIV treating physicians:* M.J.H. Pronk*, H.S.M. Ammerlaan. *HIV nurse consultants:* E.M.H.M. Korsten-Vorstermans, E.S. de Munnik. *HIV clinical virologists/chemists:* A.R. Jansz, J. Tjhie, M.C.A. Wegdam, B. Deiman, V. Scharnhorst. **Emma Kinderziekenhuis:** *HIV nurse consultants:* A. van der Plas, A.M. Weijsenfeld. **Erasmus Medisch Centrum, Rotterdam:** *HIV treating physicians:* M.E. van der Ende*, T.E.M.S. de Vries-Sluijs, E.C.M. van Gorp, C.A.M. Schurink, J.L. Nouwen, A. Verbon, B.J.A. Rijnders, H.I. Bax, R.J. Hassing, M. van der Feltz. *HIV nurse consultants:* N. Bassant, J.E.A. van Beek, M. Vriesde, L.M. van Zonneveld. *Data collection:* A. de Oude-Lubbers, H.J. van den Berg-Cameron, F.B. Bruinsma-Broekman, J. de Groot, M. de Zeeuw-de Man, M.J. Broekhoven-Kruijne. *HIV clinical virologists/chemists:* M. Schutten, A.D.M.E. Osterhaus, C.A.B. Boucher. **Erasmus Medisch Centrum–Sophia, Rotterdam:** *HIV treating*

physicians: G.J.A. Driessen, A.M.C. van Rossum. *HIV nurse consultants:* L.C. van der Knaap, E. Visser. **Flevoziekenhuis, Almere:** *HIV treating physicians:* J. Branger*. *HIV nurse consultant and data collection:* C.J.H.M. Duijf-van de Ven. **HagaZiekenhuis, Den Haag:** *HIV treating physicians:* E.F. Schippers*, C. van Nieuwkoop, R.W. Brimicombe. *HIV nurse consultants:* J.M. van IJperen. *Data collection:* G. van der Hut. *HIV clinical virologist/chemist:* P.F.H. Franck. **HIV Focus Centrum (DC Klinieken):** *HIV treating physicians:* A. van Eeden*. *HIV nurse consultants:* W. Brokking, M. Groot. *HIV clinical virologists/chemists:* M. Damen, I.S. Kwa. **Isala Klinieken, Zwolle:** *HIV treating physicians:* P.H.P. Groeneveld*, J.W. Bouwhuis. *HIV nurse consultants:* J.F. van den Berg, A.G.W. van Hulzen. *Data collection:* G.L. van der Bliek, P.C.J. Bor. *HIV clinical virologists/chemists:* P. Bloembergen, M.J.H.M. Wolfhagen, G.J.H.M. Ruijs. **Kennemer Gasthuis, Haarlem:** *HIV treating physicians:* S.F.L. van Lelyveld*, R. Soetekouw. *HIV nurse consultants:* N. Hulshoff, L.M.M. van der Prijt, M. Schoemaker. *Data collection:* N. Bermon. *HIV clinical virologists/chemists:* W.A. van der Reijden, R. Jansen, B.L. Herpers, D.Veenendaal. **Leids Universitair Medisch Centrum, Leiden:** *HIV treating physicians:* F.P. Kroon*, S.M. Arend, M.G.J. de Boer, M.P. Bauer, H. Jolink, A.M. Vollaard. *HIV nurse consultants:* W. Dorama, C. Moons. *HIV clinical virologists/chemists:* E.C.J. Claas, A.C.M. Kroes. **Maasstad Ziekenhuis, Rotterdam:** *HIV treating physicians:* J.G. den Hollander*, K. Pogany. *HIV nurse consultants:* M. Kastelijns, J.V. Smit, E. Smit. *Data collection:* M. Bezemer, T. van Niekerk. *HIV clinical virologists/chemists:* O. Pontesilli.

Maastricht UMC+, Maastricht: *HIV treating physicians:* S.H. Lowe*, A. Oude Lashof, D. Posthouwer. *HIV nurse consultants:* R.P. Ackens, J. Schippers, R. Vergoossen. *Data collection:* B. Weijenberg Maes. *HIV clinical virologists/chemists:* P.H.M. Savelkoul, I.H. Loo. **MC Zuiderzee, Lelystad:** *HIV treating physicians:* S. Weijer*, R. El Moussaoui. *HIV Nurse Consultant:* M. Heitmuller. *Data collection:* M. Heitmuller. **Medisch Centrum Alkmaar:** *HIV treating physicians:* W. Kortmann*, G. van Twillert*, J.W.T. Cohen Stuart, B.M.W. Diederen. *HIV nurse consultant and data collection:* D. Pronk, F.A. van Truijen-Oud. *HIV clinical*

virologists/chemists: W. A. van der Reijden, R. Jansen. **Medisch Centrum Haaglanden, Den Haag:** *HIV treating physicians:* E.M.S. Leyten*, L.B.S. Gelinck. *HIV nurse consultants:* A. van Hartingsveld, C. Meerkerk, G.S. Wildenbeest. *HIV clinical virologists/chemists:* J.A.E.M. Mutsaers, C.L. Jansen. **Medisch Centrum Leeuwarden, Leeuwarden:** *HIV treating physicians:* M.G.A.van Vonderen*, D.P.F. van Houte. *HIV nurse consultants:* K. Dijkstra, S. Faber. *HIV clinical virologists/chemists:* J Weel. **Medisch Spectrum Twente, Enschede:** *HIV treating physicians:* G.J. Kootstra*, C.E. Delsing. *HIV nurse consultants:* M. van der Burg-van de Plas, H. Heins. *Data collection:* E. Lucas. **Onze Lieve Vrouwe Gasthuis, Amsterdam:** *HIV treating physicians:* K. Brinkman*, P.H.J. Frissen, W.L. Blok, W.E.M. Schouten, G.E.L. van den Berk. *HIV nurse consultants:* A.S. Bosma, C.J. Brouwer, G.F. Geerders, K. Hoeksema, M.J. Kleene, I.B. van der Meché, A.J.M. Toonen, S. Wijnands. *HIV clinical virologists/chemists:* M.L. van Ogtrop, R. Jansen. **Radboud UMC, Nijmegen:** *HIV treating physicians:* P.P. Koopmans, M. Keuter, A.J.A.M. van der Ven, H.J.M. ter Hofstede, A.S.M. Dofferhoff, R. van Crevel. *HIV nurse consultants:* M. Albers, M.E.W. Bosch, K.J.T. Grintjes-Huisman, B.J. Zomer. *HIV clinical virologists/chemists:* F.F. Stelma. *HIV clinical pharmacology consultant:* D. Burger. **Rijnstate, Arnhem:** *HIV treating physicians:* C. Richter*, J.P. van der Berg, E.H. Gisolf. *HIV nurse consultants:* G. ter Beest, P.H.M. van Bentum, N. Langebeek. *HIV clinical virologists/chemists:* R. Tiemessen, C.M.A. Swanink. **Sint Lucas Andreas Ziekenhuis, Amsterdam:** *HIV treating physicians:* J. Veenstra*, K.D. Lettinga. *HIV nurse consultants:* M. Spelbrink, H. Sulman. *Data collection:* M. Spelbrink, E. Witte. *HIV clinical virologists/chemists:* M. Damen, P.G.H. Peerbooms. **Slotervaartziekenhuis, Amsterdam:** *HIV treating physicians:* J.W. Mulder, S.M.E. Vrouenraets, F.N. Lauw. *HIV nurse consultants:* M.C. van Broekhuizen, H. Paap, D.J. Vlasblom. *Data collection:* E. Oudmaijer Sanders. *HIV clinical virologists/chemists:* P.H.M. Smits, A.W. Rosingh. **Stichting Medisch Centrum Jan van Goyen, Amsterdam:** *HIV treating physicians:* D.W.M. Verhagen. *HIV nurse consultants:* J. Geilings. **St Elisabeth Ziekenhuis, Tilburg:** *HIV treating physicians:* M.E.E. van Kasteren*, A.E. Brouwer. *HIV nurse consultants and data collection:* B.A.F.M. de Kruijf-van de Wiel, M. Kuipers, R.M.W.J.

Santegoets, B. van der Ven. *HIV clinical virologists/chemists:* J.H. Marcelis, A.G.M. Buiting, P.J. Kabel. **Universitair Medisch Centrum Groningen, Groningen:** *HIV treating physicians:* W.F.W. Bierman*, H.G. Sprenger, E.H. Scholvinck, S. van Assen, K.R. Wilting, Y. Stienstra. *HIV nurse consultants:* H. de Groot-de Jonge, P.A. van der Meulen, D.A. de Weerd. *HIV clinical virologists/chemists:* H.G.M. Niesters, A. Riezebos-Brilman, C.C. van Leer-Buter. **Universitair Medisch Centrum Utrecht, Utrecht:** *HIV treating physicians:* A.I.M. Hoepelman*, M.M.E. Schneider, T. Mudrikova, P.M. Ellerbroek, J.J. Oosterheert, J.E. Arends, R.E. Barth, M.W.M. Wassenberg. *HIV nurse consultants:* D.H.M. van Elst-Laurijssen, L.M. Laan, E.E.B. van Oers-Hazelzet, J. Patist, S. Vervoort, *Data collection:* H.E. Nieuwenhuis, R. Frauenfelder. *HIV clinical virologists/chemists:* R. Schuurman, F. Verduyn-Lunel, A.M.J. Wensing. **VU Medisch Centrum, Amsterdam:** *HIV treating physicians:* E.J.G. Peters*, M.A. van Agtmael, R.M. Perenboom, M. Bomers, J. de Vocht. *HIV nurse consultants:* L.J.M. Elsenburg. *HIV clinical virologists/chemists:* A.M. Pettersson, C.M.J.E. Vandenbroucke-Grauls, C.W. Ang. **Wilhelmina Kinderziekenhuis, UMCU, Utrecht:** *HIV treating physicians:* S.P.M. Geelen, T.F.W. Wolfs, L.J. Bont. *HIV nurse consultants:* N. Nauta.

Stichting HIV Monitoring *Director:* P. Reiss. *Data analysis:* D.O. Bezemer, L. Gras, A.I. van Sighem, C. Smit. *Data management and quality control:* S. Zaheri, M. Hillebregt, A. de Jong, Y. Tong. *Data monitoring:* D. Bergsma, P. Hoekstra, A. de Lang, M. Berkhout, S. Grivell, A. Jansen, M.J. Rademaker, M. Raethke. *Data collection:* L. de Groot, M. van den Akker, Y. Bakker, M. Broekhoven, E. Claessen, E. KruijneC. Lodewijk, R. Meijering, L. Munjishvili, B. Peeck, C. Ree, R. Regtop, Y. Ruijs, M. Schoorl, E. Tuijn, L. Veenenberg, T. Woudstra. *Patient registration:* B. Tuk.

CoRIS members participating in the study were: Pompeyo Viciana, José Ramón Blanco, Juan Córdoba, María Jesús Pérez-Elías, Juan González, Jesús Santos, Ignacio Santos, Marta Álvarez, Juan Luis Gómez-Sirvent, Rafael Delgado, JoseAn Iribarren, Félix Gutiérrez, Juan Berenguer, Enrique Bernal, Asunción Iborra, María Rivero, Melchor Riera, David Dalmau, José Carlos Palomares, Francesc Vidal.

Honduras cohort: Dr. Elsa Y. Palou.

Nicaraguan cohort: Dr Guillermo Porras and Dr Carlos Quant

RFVF: Sabelo Dladla, Michelle Gordon, Jane Hampton, Brent Johnson, Daniel Kuritzkes, Roma Maharaj, Darius McDaniel, Kristy Nixon, Claudia Ordonez, Melisha Pertab and Sifiso Shange provided vital assistance for the data collection and analysis.

The Lazio and Emilia Romagna Cohorts, Italy: Massimo Andreoni, Andrea Antinori, Domenico Di Carlo, Alessandra Latini, Cristina Mussini, Carlo Federico Perno, Maria Mercedes Santoro.

The findings and conclusions in this article are those of the authors and do not necessarily represent the official position of the U.S. Centers for Disease Control and Prevention. Use of trade names is for identification purposes only and does not constitute endorsement by the U.S. Centers for Disease Control.

Funding statements for contributing sites:

KR has received the Senior Research Scholar from the Thailand Research Fund

Research reported in this publication was supported by the National Institute of Allergy and Infectious Diseases of the National Institutes of Health under Award Number UM1 AI068634, UM1 AI068636 and UM1 AI106701. The content is solely the responsibility of the authors and does not necessarily represent the official views of the National Institutes of Health.

This work was partly supported by Fonds voor Wetenschappelijk Onderzoek Vlaanderen (G.06.92.14N, PDO/11). KT receives a FWO postdoctoral fellowship. We would like to thank patients and personnel from AIDS Reference Center and Laboratory Leuven.

The Swiss HIV Cohort Study is supported by the Swiss National Science Foundation (SNF grant #33CS30-134277) and the SHCS Research Foundation. The SHCS Drug Resistance Database was further supported by the Yvonne Jacob Foundation. HFG was supported by by SNF # 320030_159868 and by the University of Zurich's Clinical research Priority Program (CRPP) "Viral infectious diseases: Zurich Primary HIV Infection Study".

RFVF: We would like to express our deepest admiration and appreciation for the patients who participated in the study and the work of the Sinikithemba Clinic at McCord Hospital in Durban, South Africa for their commitment to improve patient care and support research. The tremendous contributions on the part of the counselors, medical records staff, nurses, and medical officers have been essential to the success of this study.

The Harvard/AIDS Prevention Initiative in Nigeria (APIN) prevention, treatment and care program: Participating hospitals in this study included the University College Hospital, University of Ibadan, Ibadan, Lagos University Teaching Hospital, University of Lagos, Lagos, Jos University Teaching Hospital, University of Jos, and University of Maiduguri Teaching Hospital, Maiduguri, 68Military Hospital, Lagos, and the Nigerian Institute of Medical Research, Lagos. Sequences have been deposited in the GenBank Sequence Database under the following accession numbers: FJ931123-FJ931460.

Supplementary references

1. Gupta RK, Jordan MR, Sultan BJ, et al. Global trends in antiretroviral resistance in treatment-naive individuals with HIV after rollout of antiretroviral treatment in resource-limited settings: a global collaborative study and meta-regression analysis. *Lancet* 2012; **380**(9849): 1250-8.
2. Chan PA, Huang A, Kantor R. Low prevalence of transmitted K65R and other tenofovir resistance mutations across different HIV-1 subtypes: implications for pre-exposure prophylaxis. *J Int AIDS Soc* 2012; **15**(2): 17701.
3. Hirsch MS, Brun-Vezinet F, Clotet B, et al. Antiretroviral drug resistance testing in adults infected with human immunodeficiency virus type 1: 2003 recommendations of an International AIDS Society-USA Panel. *Clinical infectious diseases : an official publication of the Infectious Diseases Society of America* 2003; **37**(1): 113-28.
4. Parikh UM, Barnas DC, Faruki H, Mellors JW. Antagonism between the HIV-1 reverse-transcriptase mutation K65R and thymidine-analogue mutations at the genomic level. *The Journal of infectious diseases* 2006; **194**(5): 651-60.
